# Investigating cellular and molecular mechanisms of neurogenesis in *Capitella teleta* sheds light on the ancestor of Annelida

**DOI:** 10.1186/s12862-020-01636-1

**Published:** 2020-07-14

**Authors:** A. Sur, A. Renfro, P. J. Bergmann, N. P. Meyer

**Affiliations:** Department of Biology, Clark University, 950 Main Street, Worcester, MA 01610 USA

**Keywords:** Neurogenesis, Neural precursor cells, *Capitella teleta*, Annelida, Gene-regulatory network, Spiralia

## Abstract

**Background:**

Diverse architectures of nervous systems (NSs) such as a plexus in cnidarians or a more centralized nervous system (CNS) in insects and vertebrates are present across Metazoa, but it is unclear what selection pressures drove evolution and diversification of NSs. One underlying aspect of this diversity lies in the cellular and molecular mechanisms driving neurogenesis, i.e. generation of neurons from neural precursor cells (NPCs). In cnidarians, vertebrates, and arthropods, homologs of SoxB and bHLH proneural genes control different steps of neurogenesis, suggesting that some neurogenic mechanisms may be conserved. However, data are lacking for spiralian taxa.

**Results:**

To that end, we characterized NPCs and their daughters at different stages of neurogenesis in the spiralian annelid *Capitella teleta*. We assessed cellular division patterns in the neuroectoderm using static and pulse-chase labeling with thymidine analogs (EdU and BrdU), which enabled identification of NPCs that underwent multiple rounds of division. Actively-dividing brain NPCs were found to be apically-localized, whereas actively-dividing NPCs for the ventral nerve cord (VNC) were found apically, basally, and closer to the ventral midline. We used lineage tracing to characterize the changing boundary of the trunk neuroectoderm. Finally, to start to generate a genetic hierarchy, we performed double-fluorescent in-situ hybridization (FISH) and single-FISH plus EdU labeling for neurogenic gene homologs. In the brain and VNC, *Ct-soxB1* and *Ct-neurogenin* were expressed in a large proportion of apically-localized, EdU^+^ NPCs. In contrast, *Ct-ash1* was expressed in a small subset of apically-localized, EdU^+^ NPCs and subsurface, EdU^−^ cells, but not in *Ct-neuroD*^+^ or *Ct-elav1*^+^ cells, which also were subsurface.

**Conclusions:**

Our data suggest a putative genetic hierarchy with *Ct-soxB1* and *Ct-neurogenin* at the top, followed by *Ct-ash1*, then *Ct-neuroD*, and finally *Ct-elav1*. Comparison of our data with that from *Platynereis dumerilii* revealed expression of *neurogenin* homologs in proliferating NPCs in annelids, which appears different than the expression of vertebrate *neurogenin* homologs in cells that are exiting the cell cycle. Furthermore, differences between neurogenesis in the head versus trunk of *C. teleta* suggest that these two tissues may be independent developmental modules, possibly with differing evolutionary trajectories.

## Background

Early in development, a subset of ectodermal cells are specified as neuroectodermal. These neural precursor cells (NPCs) proliferate and asymmetrically divide to generate the full complement of neurons and glia of the nervous system — a process termed neurogenesis. How neurogenesis evolved across animal groups still remains ambiguous. In some clades, proliferative NPCs are distributed throughout the ectoderm, as in cnidarians [1, 2] and hemichordates [3, 4]. In other groups with a more centralized nervous system (CNS) such as arthropods, and vertebrates, NPCs are confined to a region of ectoderm, the neuroectoderm [5–15]. In vertebrates and non-insect arthropods (e.g. crabs, spiders and millipedes), NPCs remain localized to the apical surface of the ectoderm and proliferate [5, 10, 12, 14, 15]. However, notably in insects and onychophorans, the dividing neuroblasts become internalized [11, 13, 16–19]. The proliferative capacity of NPCs and the fate of the neurons and glia they generate is regulated by intrinsic transcription factors as well as extrinsic signals that together constitute a neurogenic gene regulatory network (GRN). Changes to neurogenic GRNs likely contributed to the diversity of nervous systems present in various taxa today. Understanding the cellular and molecular mechanisms underlying NPC regulation in different taxa will enable us to better understand the evolution of neurogenesis.

Our most in-depth understanding of neurogenic GRNs comes mainly from studies of vertebrates and the fruit fly, *Drosophila melanogaster* — two evolutionarily distant groups*.* These studies show that transcriptional regulators belonging to the SoxB family and basic-helix-loop-helix (bHLH) type A family (e.g., Achaete-Scute homologs, Neurogenin, and Atonal), regulate various aspects of neurogenesis [9, 20–27]. Generally, both spatiotemporal expression as well as functional analyses of SoxB1 homologs in chordates and *D. melanogaster* indicate a role in maintaining NPCs in an undifferentiated state [5, 28–45]. However, SoxB1 homologs have been shown to possess additional roles in terminal differentiation and neural subtype specification in mice [46–49], *D. melanogaster* [38, 50], and *Caenorhabditis elegans* [51].

Achaete-Scute homologs are deployed in slightly different ways in vertebrates and insects. In chick and mice, Achaete-Scute homologs induce cell cycle exit, migration of neuronal cells, and neuronal differentiation [5, 22]. In contrast, *D. melanogaster* Achaete-Scute family members (i.e., Achaete, Scute, and Lethal of Scute) help specify neural fate and induce internalization of neuroblasts, which divide later [52–55]. Moreover, recent reports from mice and *D. melanogaster* embryos as well as human neural stem cell cultures revealed additional and unexpected functions of Achaete-Scute homologs (i.e. Ascl1 and Asense) in promoting NPC proliferation by directly activating cell-cycle regulators [24, 56, 57]. Reports from the cnidarian *Nematostella vectensis* [1, 2, 58, 59] and the acoel *Symsagittifera roscoffensis* [60, 61] reveal that some *achaete-scute* gene homologs are expressed in differentiating neural cells. Overall, new data and taxonomic diversity of study animals is highlighting the multifaceted functions of these core transcription factor complexes thereby complicating assumptions about their ancestral roles.

A major reason for such ambiguous understanding of the evolution of neurogenesis is a lack of information from the third major bilaterian clade, Spiralia, which includes annelids, mollusks, nemerteans, and platyhelminthes. Only by contrasting spiralian neurogenesis with our current knowledge from chordates, arthropods, and cnidarians can we reconstruct how bilaterian nervous systems evolved. Our current understanding of spiralian neurogenesis is derived mostly from *Platynereis dumerilii* [62–65] and *Capitella teleta* [66, 67], which belong to the two major annelid clades, Errantia and Sedentaria, respectively [68, 69]. In *P. dumerilii*, an apical, proliferating cell population in the trunk neuroectoderm that expressed *Pdu-ngn* and *Pdu-ash1* was identified as distinct from basal cell populations expressing neural differentiation markers such as *Pdu-synaptotagmin* and *Pdu-collier* [63, 65]. In the head of *C. teleta*, single neural cells ingress from localized areas in the anterior ectoderm that express *Ct-ash1* to generate the brain [66, 67], which is similar to arthropod neurogenesis. However, unlike insects, in *C. teleta*, cell divisions predominantly take place in superficial neuroectodermal cells and not in neural cells that have ingressed. Internalized brain cells express homologs of the neuronal markers *elav* (*Ct-elav1*) and *synaptotagmin I* (*Ct-syt1*) [66, 70]. In the developing ventral nerve cord (VNC), *Ct-soxB1* and *Ct-ngn* are amongst the first genes expressed in the neuroectoderm followed in sequence by *Ct-ash1*, *Ct-neuroD*, *Ct-elav1*, and finally *Ct-syt1* [67]. However, the spatial and temporal origin of NPCs in the VNC and the gene regulatory network controlling neurogenesis in the brain and VNC in *C. teleta* are largely unknown.

Therefore, we focused on correlating cellular behavior with gene expression to start generating a preliminary neurogenic GRN in the annelid *C. teleta*. We characterized the position and boundaries of the neuroectoderm in the trunk of *C. teleta* during development as well as the spatiotemporal position of proliferative cells in the ventral neuroectoderm using a combination of labeling with fluorescent dye and pulse-chase experiments with two different thymidine analogs. We also characterized gene co-expression in dividing NPCs and differentiating neurons in the brain and VNC by single and double fluorescent in situ hybridization (FISH) and labeling with thymidine analogs in order to better understand the molecular progression of neurogenesis.

## Results

### An apical repertoire of proliferative NPCs in the head gives rise to the brain

To better understand whether brain NPCs in *C. teleta* remain apically-localized and undergo multiple rounds of division, we performed a series of labeling experiments with thymidine analogs. Cell proliferation profiles in the anterior neuroectoderm from stages 3–6 were examined using a 30-min pulse of EdU followed by fixation. These data confirmed previous results [66, 67] showing that proliferating cells in S-phase are apically-localized in the anterior neuroectoderm (Additional File 1: Fig. S1a–d). At stage 6, a few EdU^+^ cells were also detected outside the basal boundary of the forming brain (Additional File 1: Fig. S1d; arrows). These EdU^+^ cells outside the basal edges of the brain have a different cellular shape and nuclear size, and were previously hypothesized to be head mesodermal cells derived from the 3a blastomere based on fate-mapping studies [66, 71]. The number and proportion of EdU^+^ cells relative to the total number of cells (Hoechst^+^) increased steadily and significantly from stages 3–5 and then drastically decreased at stage 6 (ANOVA, F_3,75_ = 64.68, *p* < 0.001) (Additional File 1: Fig. S1h–j), indicating that cell proliferation declines at later stages. 45-min EdU labeling followed by anti-phospho-Histone H3 (PH3) immunostaining to label mitotic cells [72] further confirmed that proliferating cells are restricted to the apical surface of the anterior neuroectoderm (Additional File 1: Fig. S1e–g).

Next, we examined how NPCs that were proliferative at stage 4 contributed to the brain by performing an EdU pulse-chase experiment. Animals were pulsed with EdU for 30 min at stage 4 followed by a 3-h (h) thymidine chase, incubation in seawater till 48 h, and fixation at seven time-intervals as indicated (Additional File 2: Fig. S2a, b). We quantified the number and proportion of EdU^+^ cells using 30 μm × 30 μm wide × 10 μm deep regions of interest (ROIs) across different depths of the head (Additional File 2: Fig. S2c) at different time-points and tested for differences using mixed effects modeling (Additional File 8: Table S1, S2). Our mixed effects models fit the data well, explaining between 88.1 and 96.2% of variation in the response variables for the head. The fixed effects also explained substantial amounts of total variation in the head counts (63.5–80.8%; Additional File 8: Table S1). In the head, cell depth, nested in individual animal accounted for the most random effects variation, with the side of the embryo and mother each individual came from accounting for less and comparable variation. Individual animal variation accounted for very little variation.

At 0 h after the EdU pulse, the majority of EdU^+^ cells were apically-localized (0–10 μm depth; Additional File 2: Fig. S2b, 0 h). From 3 to 20 h after the EdU pulse (stages 4–5), the number and proportion of apically-localized (0–10 μm depth, ROI 1) EdU^+^ cells remained relatively constant, but the number and proportion of EdU^+^ cells in the more basal regions of the developing brain (30–40 μm depth, ROI 4) increased with time (Additional File 2: Fig. S2b 3–20 h, d–f). By 36–48 h after pulse (stage 6), a significantly larger proportion of EdU^+^ cells occupied the intermediate (i.e. just below the surface, ROI 2 and 3) and basal regions of the brain relative to the apical surface (ROI 4, Additional File 2: Fig. S2b 36–48 h, d–f). From 0 to 48 h, the total number of EdU^+^ cells across all ROIs increased almost five-fold (0 h, stage 4 telotroch, 97.5 ± 0.910 S.E.M.; 48 h, stage 6 mid, 499.2 ± 2.001 S.E.M.), suggesting a doubling rate of ~ 24 h in the anterior neuroectoderm (Additional File 2: Fig. S2d; data not shown; Additional File 3: Table S7). However, the total number of nuclei in anterior neuroectoderm across the same timeframe (0–48 h) only doubled (0 h, 480.3 ± 2.273 S.E.M.; 48 h, 1005.7 ± 2.753 S.E.M.) across ROIs 1–4, indicating that cells not labeled with EdU at stage 4 may include non-dividing cells and/or more slowly dividing cells. Doubling rate was calculated using EdU and Hoescht counts across ROIs 1–4 although counts and proportions from only ROIs 1 and 4 are plotted in Additional File 2: Fig. S2d–f (See Materials and Methods). Raw data for EdU and Hoescht counts across all ROIs are available in Additional File 3: Table S7. Overall, throughout the entire chase duration, a relatively constant number of EdU^+^ cells remained apical while increasing numbers of EdU^+^ cells were found in the brain (Additional File 2: Fig. S2b, d), suggesting that some NPCs remain apically-localized from stages 4–6 while undergoing multiple rounds of division, which we refer to as “dedicated NPCs”. Some of their daughter cells are then internalized to form the brain.

**Fig. 1:**
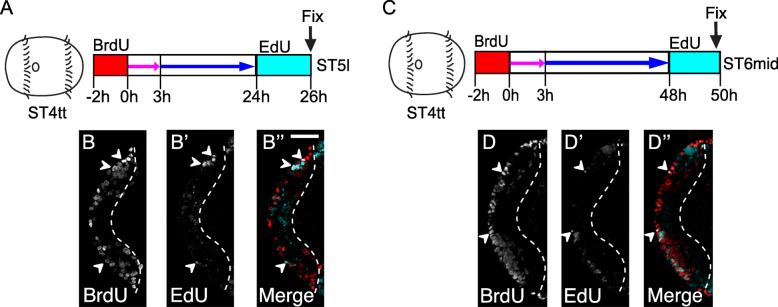
Dividing NPCs undergo multiple rounds of division while maintaining apical contact. **a, c** Schematic showing stage 4 telotroch animals exposed to BrdU (red) for 2 h followed by a 3 h thymidine chase (pink arrow) and sea-water incubation (blue arrow) until 24 h **a** and 48 h **c** respectively before a 2 h EdU (cyan) pulse. **b, b′, b**”, **d, d’, d**”) Confocal micrographs of BrdU pulse-chase-wait-EdU experiments showing BrdU (red) and EdU (cyan) after chase for 24 h **b–b**″ and 48 h **d–d**”. Panels **b, b′, b**″, **d, d’, d**” represent ventral head views showing single BrdU^+^ (**b, d**), EdU^+^ (**b′, d’**) and EdU/BrdU dual-labeled cells (**b″, d”**). Closed arrowheads show EdU/BrdU dual-labeled cells at the apical surface of the anterior neuroectoderm. Dashed lines indicate the basal boundaries of the developing brain. The channel visualized is indicated on the lower left corner of each figure panel. ST4tt: Stage 4 telotroch, ST5l: Stage 5 late, ST6mid: stage 6 mid. Scale bar: 25 μm

To test for the presence of dedicated NPCs, we performed BrdU pulse-chase-wait-EdU sequential labeling (Fig. 1). Animals were initially pulsed with BrdU for 2 h at stage 4 telotroch, chased with thymidine for 3 h and incubated in seawater for a total of 24 h or 48 h before being exposed to a second pulse with EdU for 2 h, i.e., at stage 5 or 6 (Fig. 1a, c). This experimental design allowed us to identify cells that were in S phase at two separate time intervals and could therefore be dedicated NPCs. After both intervals, EdU/BrdU dual-labeled nuclei were present, but only in the surface neuroectoderm (Fig. 1b″, d″; closed arrowheads). Basally-localized nuclei in the developing brain were only labeled with BrdU^+^, suggesting that these cells were derived from dividing cells labeled at stage 4 (Fig. 1b, b″, d, d″). No dual-labeled nuclei or solely EdU^+^ nuclei were detected below the surface neuroectoderm at 24 or 48 h (Fig. 1b′, b″, d′, d″). These data support our hypothesis [66] that dedicated NPCs maintain apical contact with the surface neuroectoderm in the head, while their daughters contribute neural cells to the brain.

### Formation of the ventral trunk neuroectoderm and initiation of trunk neurogenesis

**Fig. 2:**
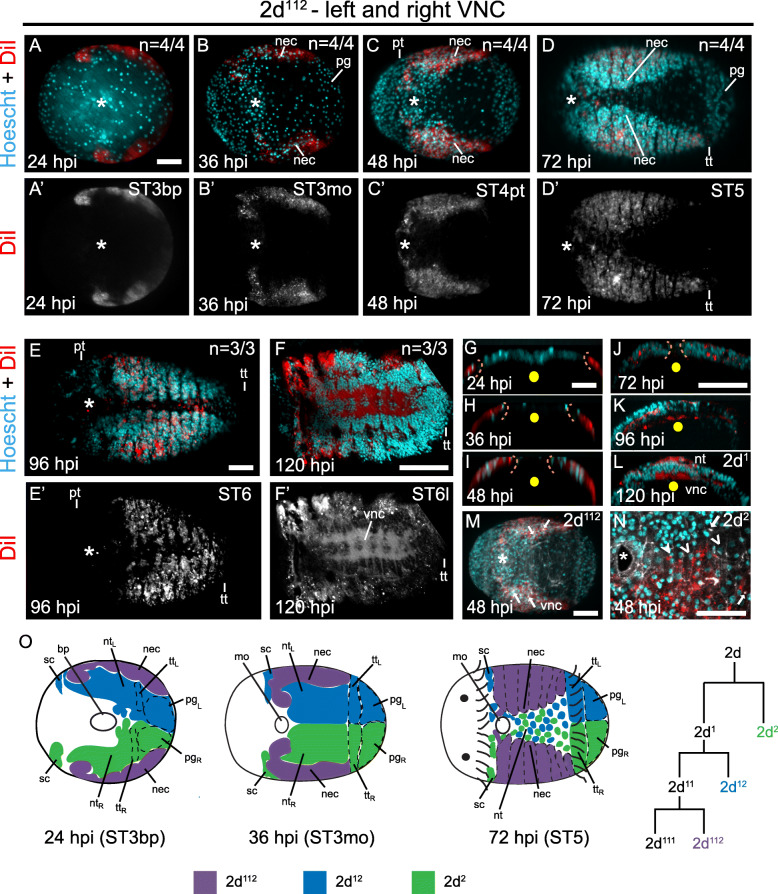
The presumptive neuroectoderm of the VNC is derived from sub-clones of the 2d^112^ blastomere. (**a–f**′) Apotome images of DiI injected animals from stages 3–6 shown in ventral view. Top rows (**a, b, c, d, e, f**) show merged channels for nuclear stain (cyan) and DiI (red) while bottom row (**a**′, **b**′, **c**′, **d**′, **e**′ **f**′) indicates only DiI labeling in black and white. Panels **a–e**’ are superficial, panels f and f' indicate a subsurface projection showing VNC ganglia. In panels f and f', DiI labeling in the neurites is brighter than other tissues as labeling in the cell bodies become patchier after fixation. (**g–l**) Orthogonal sections through the anterior half of the ventral trunk neuroectoderm showing apical (up) and basal (down) boundaries of DiI labeled patches (red) and nuclear stain (cyan). Panel **l** highlights an orthogonal view of a 2d^1^ injected embryo grown for 120 hpi (stage 6 late) showing neurotroch (nt) on the surface and VNC subsurface. (**m, n**) Ventral views of DiI injected animals at 48 hpi (stage 4) with cell outlines marked with F-actin (white) for a 2d^112^ injected animal **m** and a 2d^2^ injected animal **n**. Arrows in m and n indicate F-actin bundles showing apical constriction. Closed arrowheads in panel n shows a DiI^+^ cell intercalated with DiI^−^ cells denoted by open arrowheads. **o** Diagram showing the different tissues contributed by each of the 2d daughters at different developmental stages encompassing neurogenesis. The length of time grown is indicated as hours post-injection (hpi) on the lower left corner of each figure panel. Asterix denotes the position of the mouth. In panels **a–f**′, the number of animals evaluated at each stage after injection is shown at the upper right corner. The corresponding stages of each animal is indicated on the upper right corner of the lower panels a’–f′. In all orthogonal views **g–l**, apical is up and basal is down and the yellow dot shows the position of the ventral midline. Orange dashed line indicated the leading edge of the DiI^+^ trunk neuroectoderm. Prototroch (pt) and telotroch (tt) are indicated by dashes. bp: blastopore, nt: neurotroch, nt_L_: left neurotroch, nt_R_: right neurotroch, mo: mouth, sc: sensory cell, nec: neuroectoderm, vnc: ventral nerve cord, pt.: prototroch, pt_L_: left prototroch, pt_R_: right prototroch tt: telotroch, tt_L_: left telotroch, tt_R_: right telotroch, pg: pygidium, pg_L_: left pygidium, pg_R_: right pygidium, ST3bp: Stage 3 blastopore, ST3mo: Stage 3 mouth, ST6l: Stage 6 late. Scale bar: 50 μm

As neurogenesis of the VNC is not well understood in *C. teleta*, we first characterized the ventral boundaries of the neuroectoderm in the trunk by lineage tracing. We injected the lipophilic dye DiI into daughters of the 2d somatoblast, 2d^112^ (Fig. 2, Additional File 5: Movie S1) and 2d^1^, 2d^11^, and 2d^2^ (Additional File 4: Fig. S3, Additional File 6: Movie S2), and tracked the labeled daughters from gastrulation through formation of the VNC. Previous work showed that descendants of 2d^112^ form most of the ectoderm in the trunk including the VNC, while descendants of 2d^2^ and 2d^12^ form the right and left sides, respectively, of the neurotroch (ventral midline ciliary band), telotroch (posterior ciliary band), and pygidial ectoderm [71, 73]. By comparing the relative position of DiI^+^ cells that contributed to the neurotroch (2d^2^) versus DiI^+^ cells that formed the neuroectoderm (2d^11^ and 2d^112^) or neuroectoderm plus left neurotroch (2d^1^), we were able to identify the ventral edge of the forming neuroectoderm at each stage of development.

Towards the end of gastrulation (24 h post injection, hpi; stage 3 blastopore), cells in the trunk ectoderm are segregated based on their fate and the tissues to which they contribute. The vegetal-most edge of the trunk ectoderm, which presumably represents the vegetal-most edge of the presumptive neuroectoderm, is lateral and slightly vegetal as indicated by DiI^+^ daughters of 2d^112^ (Fig. 2a, a’, g, o), 2d^11^ (Additional File 4: Fig. S3a, a’), and 2d^1^ (Additional File 4: Fig. S3e, e’) blastomeres. During gastrulation, the vegetal edge of the presumptive neuroectoderm moves vegetally and once gastrulation is completed, continues to move ventrally (Fig. 2, Additional File 4: Fig. S3, Additional File 5: Movie S1, Additional File 6: Movie S2) until the anterior ganglia of the VNC become visible at ~ 72 hpi (stage 5; Fig. 2d, d’, o). The 2d^112^ -derived DiI^+^ ectoderm extends all the way to the dorsal side of the animal from 24 to 120 hpi (stage 3 blastopore to stage 6 late; data not shown) [73].

At 24 hpi (stage 3 blastopore), DiI^+^ cells descended from 2d^1^ and 2d^2^ blastomeres, which contribute to the left and right sides of the neurotroch, respectively, are vegetally positioned and occupy the region between the blastopore and the 2d^112^-derived presumptive neuroectoderm (compare Additional File 4: Fig. S3e, e’, i, i′ with Fig. 2a, a’). During gastrulation, the presumptive neurotroch cells always remain more vegetal relative to the presumptive neuroectoderm. At stage 4 (48 hpi), the presumptive neurotroch appears to be 10–12 nuclei wide spanning the entire mid-ventral region between the left and right neuroectoderm (Additional File 4: Fig. S3g, g’, k, k′). At this time, the neurotrochal cells are much larger than the presumptive neuroectodermal cells as indicated by the phallicidin-labeled cell outlines (data not shown). The neurotrochal cells change shape from stages 5–6 (72–96hpi) and appear to intercalate with one another from left to right (i.e., undergo convergent extension), resulting in a longer and narrower neurotroch (Additional File 4: Fig. S3h, h′, l, l’; Additional File 5: Movie S1, Additional File 6: Movie S2). The distance between adjacent neurotrochal nuclei decreases from stage 4 (48 hpi) to stage 5 (72 hpi) (compare Additional File 4: Fig. S3l with k), and cell intercalation of daughters of 2d^1^ and 2d^2^ can be seen as a row of alternating DiI^+^ and DiI^−^ cells along the ventral midline by late stage 5 (72 hpi; Additional File 4: Fig. S3h, h′, l, l’, arrowheads). While the neurotrochal cells are intercalating, the neuroectodermal cells (daughters of 2d^11^ and 2d^112^) continue to move ventrally and begin to undergo segmentation adjacent to the ventral midline (Fig. 2d, d’; Additional File 4: Fig. S3d, d’, h, h′).

As the VNC forms below the surface trunk ectoderm, we also assessed how the trunk neuroectoderm develops along the apical-basal axis by lineage tracing the 2d^112^ blastomere. From late gastrulation (stage 3 blastopore; 24 hpi) to stage 3 mouth (36 hpi), the vegetal-most DiI^+^ domain derived from 2d^112^ appears to be a simple epithelium as most of the nuclei are apically-localized with only a few basally-shifted nuclei (Fig. 2b, g, h). However, from stage 4 prototroch (48 hpi) onwards, more subsurface DiI^+^ nuclei were detected, likely indicating cell internalization (Fig. 2c, i-l). During this time, apical spots of concentrated F-actin were detected using phallacidin, suggesting the presence of bottle cells undergoing apical constriction (Fig. 2m, n; arrow). However, at later stages (e.g., stage 5, 72 hpi), once the muscles begin to develop, it becomes very difficult to detect cell outlines using phallacidin as this strongly labels all muscle fibers. Therefore, without a better marker of cell membranes, we were not able to deduce the mechanisms of cell internalization in the trunk neuroectoderm at later developmental stages in *C. teleta*. During this time, VNC ganglia are first detected in segments 2–7 (Fig. 2d, d’; Additional File 4: Fig. S3d, d’, h, h′). The VNC keeps adding more ganglia posteriorly as the animal elongates. By stage 6 (96 hpi), VNC ganglia in segments 8–10 are also visible (Fig. 2e–f). Late stage 6 (120 hpi) onwards, DiI^+^ neurites comprising multiple longitudinal strands as well as connectives and commissures of the VNC can be visible below the surface (Fig. 2f, f’, l). During this time the neurotroch is located along the ventral midline on the apical surface (Fig. 2l).

The ventral boundaries of the presumptive trunk neuroectoderm that we identified using DiI labeling were used as a reference for subsequent experiments that examined formation of VNC NPCs and their behavior.

### Cell division patterns in the trunk neuroectoderm

**Fig. 3:**
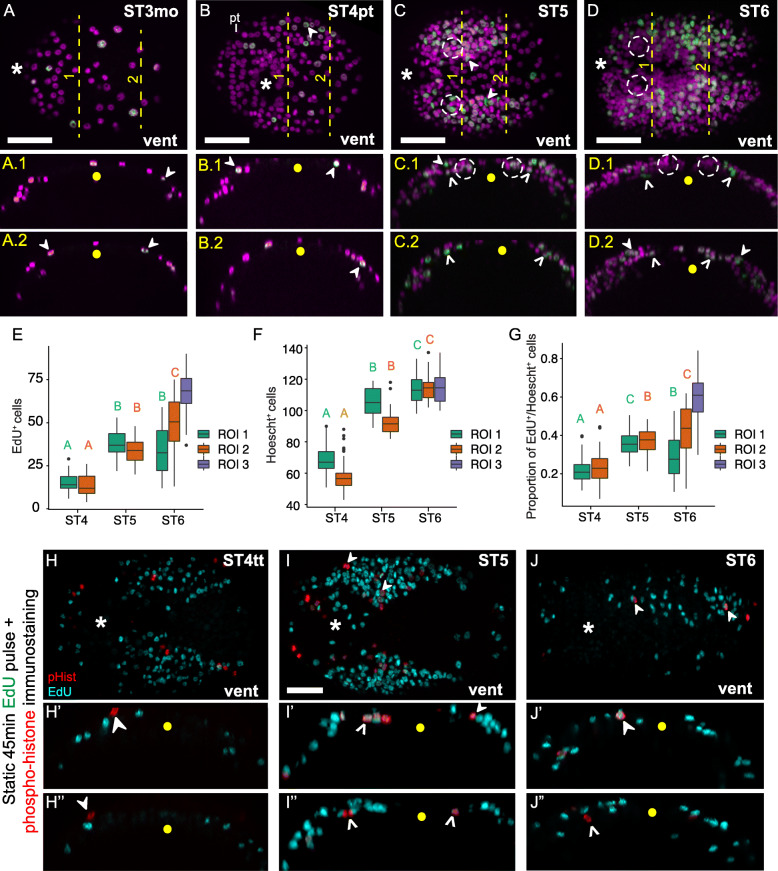
Cell proliferation profiles and architecture of the trunk neuroectoderm. **a, b, c, d** Apotome images of larval trunk neuroectoderm showing patterns of cell proliferation following static 30-min EdU (green) pulses and nuclear stain (magenta). Bottom two rows (a.1, a.2, b.1, b.2, c.1, c.2, d.1, d.2) show orthogonal projections of the trunk neuroectoderm through anterior and posterior segment groups along the yellow dashed lines in **a, b, c, d** labeled 1 and 2. Closed arrowheads in a-d.2 indicate surface EdU^+^ cells while open arrowheads indicate subsurface EdU^+^ cells within the boundaries of the presumptive neuroectoderm. **e–g** Graphs indicating comparative cell proliferation profiles between segments of the trunk across stages 4–6. The capital letters on top of the boxplots (e.g. A, B etc.) indicate statistical significance for comparison of individual segment groups (ROIs) across stages based on our mixed effects model analysis. The upper and lower bounds of the box plot indicate the 3rd and 1st quartiles while the middle line inside the boxplot indicates the median. The ends of the whiskers represent the 5th and 95th percentiles, dots represent outliers. **h–j** 45-min static EdU labeling coupled with PH3 immunostaining across stage 4 till stage 6. h', h", i', i", j' and j" represent orthogonal views of the trunk neuroectoderm through anterior (h', i', j') and posterior regions h", i", j" of the trunk. Closed arrowheads indicate PH3+ cells adjacent to another PH3+ cell or EdU^+^ cell. Open arrowheads indicate subsurface PH3+ cells in the trunk. In panels **a, b, c, d, h, i, j**, anterior is to the left and posterior to the right. The stage investigated is indicated on the upper right-hand corner, while the orientation of the animal is indicated on the lower right corner. Dashed circles in c, c.1, d, d.2 indicate presence and loss of EdU^+^ cells from segments 2–4. In orthogonal views (a.1, a.2, b.1, b.2, c.1, c.2, c.1, d.2, h′, h″, i′, i″,j’, j″), apical is up and basal is down and the yellow dot denotes the ventral midline. ST3mo: Stage 3 mouth, ST4pt: Stage 4 prototroch, ST4tt: Stage 4 telotroch. Vent, ventral, Asterisk indicates the mouth. Scale bar: 50 μm

To better understand where cells are dividing in the trunk neuroectoderm, we incubated embryos and larvae from stages 3–6 for 30 min in the thymidine analog EdU followed by immediate fixation. During gastrulation, EdU^+^ cells were scattered throughout the animal cap including the region of the presumptive neuroectoderm (data not shown). After closure of the blastopore, a few EdU^+^ cells were detected throughout the presumptive trunk neuroectoderm at stage 3 (Fig. 3a) and stage 4 (Fig. 3b, b.1, b.2; closed arrowheads). We quantified the number of EdU^+^ cells, the number of Hoechst^+^ cells, and the proportion of Hoechst^+^ cells that were also EdU^+^ within three different regions of interest (ROIs) in the trunk neuroectoderm from anterior to posterior and tested for differences using mixed effects modeling (Additional File 7: Fig. S4h, Additional File 8: Table S3, S4, see Materials and Methods). Our mixed effects model explained between 84.9 and 93.8% of variation in the response variables, with 39.3 to 84.7% of those variations explained by fixed effects (Additional File 8: Table S3). Two of the ROIs roughly corresponded to the future position (stage 4) or actual position (stages 5 and 6) of segments 2–4 (ROI 1) and 5–7 (ROI 2). ROI 3 (segments 8–10) was only scored at stage 6, once this region was present since *C. teleta* elongates posteriorly. At stage 4, EdU^+^ cells constituted ~ 20% of quantified neuroectodermal cells within ROIs 1 and 2 (Fig. 3g). From stage 4 to 5, the number and proportion of EdU^+^ cells in ROI 1 increased significantly (ST4, 15.2 ± 0.713 S.E.M.; ST5, 38.2 ± 1.053) (Fig. 3b, c, e, g) and remained constant through stage 6 (33.3 ± 2.040) (Fig. 3e, g). From stage 5 to 6 in segments 1 and 2, we observed a loss of EdU^+^ cells (Fig. 3d, d.1; compare with Fig. 3c, c.1, white dashed circles). In contrast, in ROI 2, the number of EdU^+^ cells almost doubled twice from stages 4–6 (ST4, 14.1 ± 0.847 S.E.M.; ST5, 34.1 ± 0.927; ST6, 49.6 ± 2.491; Fig. 3e, g). Overall, the total number of cells (Hoechst^+^) in the trunk neuroectoderm increased consistently from stages 4–6 in both ROIs 1 and 2 (Fig. 3f). At stage 6, the number and proportion of EdU^+^ cells in ROI 3 were higher than in ROI 1 or 2 (ST6, 67.4 ± 0.966) (Fig. 3e, g). This suggests that neurogenesis proceeds from anterior to posterior with NPCs in ROI 1 beginning to exit the cell cycle by stage 6 while NPCs in ROI 2 and 3 are still actively dividing.

At all stages examined (stages 3–6), we observed surface EdU^+^ cells in the trunk neuroectoderm (Fig. 3a–d, closed arrowheads). However, unlike in the brain, by stage 5, we also observed subsurface EdU^+^ cells in both ROI 1 (Fig. 3c, c.1, open arrowheads) and ROI 2 (Fig. 3c, c.2, open arrowheads). At stage 6, ROI 1 and 2 had more subsurface EdU^+^ cells than stage 5, while ROI 3 contained mostly apically-positioned nuclei with a few basally-shifted nuclei (Fig. 3d, d.1, d.2, open arrowheads). Cell proliferation profiles were further explored using 45-min EdU pulses followed by anti-phospho-Histone H3 (PH3) labeling at stages 4–6. At all stages examined, only a few PH3^+^ cells were detected simultaneously in the trunk neuroectoderm (Fig. 3h–j), indicating that *C. teleta* NPCs do not undergo synchronous waves of mitosis. Surface PH3^+^ cells were observed at all stages in the trunk neuroectoderm similar to the brain (Fig. 3h–j, closed arrowheads). Similar to the pattern observed with EdU, we also found subsurface PH3^+^ cells (Fig. 3i’, i, j’, j”; open arrowheads) in both anterior and posterior orthogonal sections at stages 4–6, indicating that internalized cells also undergo mitosis in the trunk neuroectoderm in *C. teleta*. Single PH3^+^ cells were often found adjacent to another PH3^+^ cell, likely indicative of telophase, or to an EdU^+^ cell, possibly indicative of adjacent NPCs (Fig. 3i–j; closed arrowheads). Assessment of spindle orientations of PH3^+^ cells in the trunk neuroectoderm revealed that mitotic spindles were largely parallel to the plane of the trunk neuroepithelium (Fig. 3i”), although oblique spindles were occasionally observed (Fig. 3j”). Mitotic spindles orientated perpendicular to the epithelial plane were never observed.

**Fig. 4:**
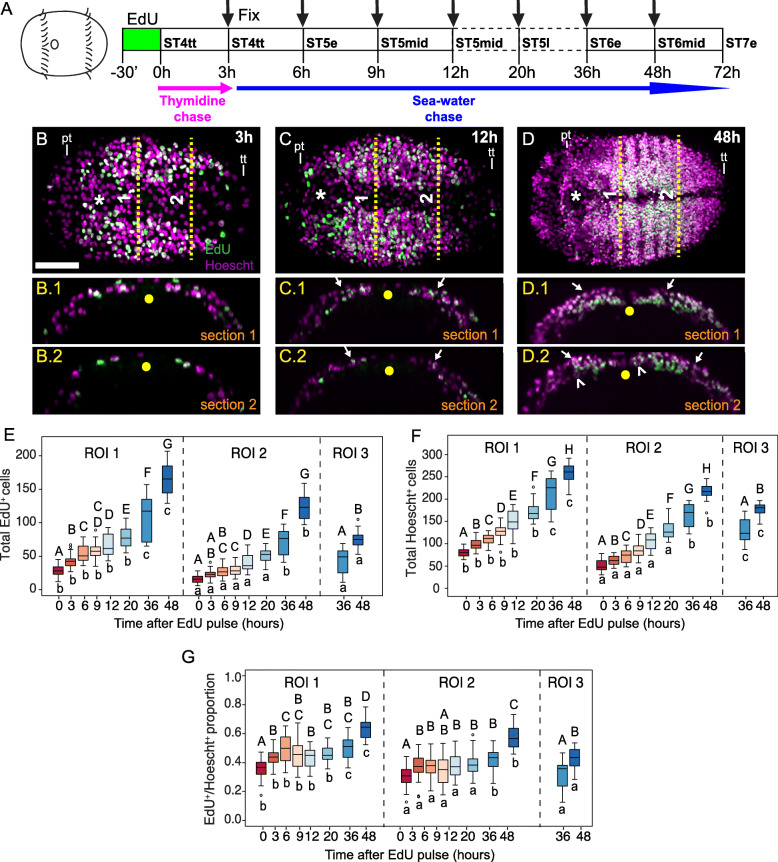
Cell-cycle kinetics and dynamics of cell proliferation in the trunk neuroectoderm. **a** Schematic showing EdU pulse chase experiment with EdU pulse at stage 4 telotroch followed by 3 h of 10 μM thymidine chase and subsequent incubation in sea-water until indicated time periods. (**b**–**d**) Ventral views **b, c, d** and orthogonal views (b.1, b.2, c.1, c.2, d.1, d.2) of larval trunk neuroectoderm at three different time intervals (3 h, 12 h and 48 h) shown labeled with EdU (green) and Hoechst (magenta). b.1, c.1 and d.1 indicate orthogonal views along the dashed line labeled “1” and b.2, c.2 and d.2 represent orthogonal views along the dashed line labeled “2” in b, c, d respectively. Arrows in c.1, c.2, d.1, d.2 indicate weakly labeled EdU^+^ cells which are generally localized on the surface trunk ectoderm while open arrowheads indicate subsurface uniformly labeled EdU^+^ cells. (**e**–**f**) Plots showing number of EdU^+^ cells **e**, all cells labeled by Hoechst **f** and proportion of EdU^+^ relative to Hoechst labeled cells **g** at different lengths of time after the chase for specific ROIs as indicated in Additional File 7: Fig. S4h. Boxplots are organized into panels by ROI and ordered by time following chase – 0 h (red) and 48 h (blue), with times post EdU pulse indicated in hours on the x-axis. Capital letters above the boxplots indicate statistical groups comparing cell counts or proportions at different times within a ROI. The lowercase letters below the boxplot indicate statistical groups comparing cell counts or proportions for different ROIs at each given chase timing. Different letters represent significant differences with *p* < 0.05 after correction for multiple comparisons. Panels b, c, d show ventral views with anterior to left and posterior to the right. In all ventral views, asterisk indicates the position of the mouth while the yellow dot in the orthogonal views (b.1, b.2, c.1, c.2, d.1, d.2) indicate the position of the ventral midline. In all orthogonal views, apical is up and basal is down. ST4tt: stage 4 telotroch, ST5e: stage 5 early, ST5mid: stage 5 middle, ST5l: Stage 5 late, ST6e: stage 6 early, ST6mid: stage 6 mid. pt.: prototroch, tt: telotroch. Scale bar: 50 μm

Next, to estimate the contribution to the VNC of cells proliferating at stage 4, we performed an EdU pulse-chase experiment starting at stage 4, once the telotroch was visible. Briefly, stage 4 larvae were pulsed with EdU for 30 min, followed by a 3 h chase in thymidine, incubation in seawater, and fixation at 3 h intervals until mid-stage 5 and then at ~ 12 h intervals until stage 7 (Fig. 4a; Additional File 7: Fig. S4a). From 0 h (stage 4 telotroch) through 12 h (mid-stage 5) after the EdU pulse, EdU^+^ nuclei were randomly scattered throughout the presumptive trunk neuroectoderm (Fig. 4b, c; Additional File 7: Fig. S4b–d). However, 36–72 h after pulse (mid-stage 6 to early stage 7), we observe more EdU^+^ cells localized in the forming ganglia of the VNC (Fig. 4d; Additional File 7: Fig. S4f, g). From 0 to 12 h after pulse (stage 4 telotroch to mid-stage 5), more surface EdU^+^ cells were observed as compared to those subsurface (Fig. 4b-c; Additional File 7: Fig. S4b–d), but as the ganglia of the VNC began to form 20–72 h after pulse (late stage 5 to early stage 7), subsurface EdU^+^ cells outnumbered surface EdU^+^ cells (Fig. 4d; Additional File 7: Fig. S4e–g).

We further quantified the number and proportion of EdU^+^ cells after pulse-chase within the same ROIs 1–3 in the trunk neuroectoderm at different time points (Additional File 7: Fig. S4h; Fig. 4e-g, Additional File 9: Table S8). In order to statistically compare counts between anterior versus posterior segments across time and to decipher trends in cellular patterns during trunk neurogenesis, we performed a mixed effects model analysis (Additional File 8: Table S1, S2), similar to the brain. Our mixed effects models explained between 85.6 and 98.2% of variation in the response variables (Additional File 8: Table S1), and a substantial amount of this variation was explained by the fixed effects (45.5–88.3%; Additional File 8: Table S1). In the trunk, mother and individual animal accounted for the plurality of variation explained by random effects, with ROI nested in individual and left versus right side accounting for less (Additional File 8: Table S2).

EdU^+^ cells constituted ~ 40% of all counted nuclei in ROI 1 and ~ 30% of all counted nuclei in ROI 2 at 0 h (Fig. 4e). The number of EdU^+^ and Hoechst^+^ cells in ROI 1 and 2 increased from 0 to 48 h after EdU pulse (Fig. 4e, f). While the numbers of EdU^+^ cells from 0 to 48 h after pulse were slightly lower in ROI 2 (0 h, stage 4 telotroch, 16.1 ± 0.965; 48 h, stage 6 mid, 124.3 ± 3.980) compared to ROI 1 (0 h, 29.0 ± 1.191 S.E.M.; 48 h, 165.6 ± 4.472), the fold increase was greater in ROI 2 (~ 8-fold increase) versus ROI 1 (~ 6-fold increase) across the same time span. In contrast, the number of Hoechst^+^ nuclei only increased 3–4 fold in ROI 1 (0 h, 80.4 ± 1.543; 48 h, 258.9 ± 4.305) and 2 (0 h, 51.1 ± 1.919; 48 h, 216.4 ± 3.316) from 0 to 48 h after EdU pulse, indicating that some Hoechst^+^/EdU^−^ cells may be dividing more slowly or may not be dividing at all (Fig. 4f). The proportion of EdU^+^ cells in both ROI 1 and 2 remained fairly constant across timepoints, except for a significant increase from 36 to 48 h after EdU pulse (Fig. 4g). These data could either indicate a progressive anterior-to-posterior exit of NPCs from the cell-cycle (as seen with static EdU labeling) and/or that the cell-cycle lengths in anterior versus posterior segment groups (ROIs 1 and 2) are not the same. Cells in ROI 3 were only scored at 36 and 48 h after EdU pulse and were found to almost double in number of EdU^+^ cells across the two time points (36 h, stage 6 early, 42.5 ± 3.890; 48 h, stage 6 mid, 75.6 ± 2.553; Fig. 4e), indicating a rapid doubling rate (i.e. ~ 12 h) in the newly formed segments 8–10.

**Fig. 5:**
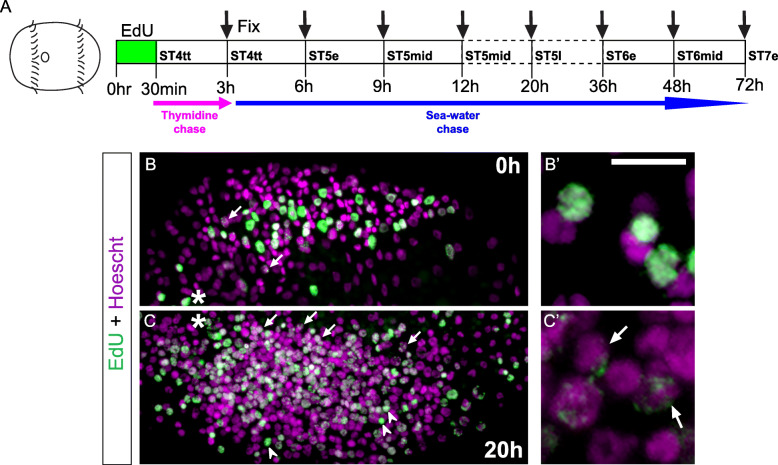
Differential EdU labeling associated with the chromatin. **a** Schematic showing EdU pulse chase experiment with EdU pulse at stage 4 telotroch followed by 3 h of 10 μM thymidine chase and subsequent incubation in ASW until indicated time periods where animals were fixed. (**b**–**c**) Close-up ventral images showing differential EdU labeling after a 20 h chase. Panels b', c' shows enlarged images of uniform **b**′ and stippled **c**′ labeling of EdU^+^ cells observed at 0 and 20 h post EdU pulse respectively. Arrows indicate probable actively dividing NPCs that exhibit weak EdU labeling due to multiple rounds of mitosis while closed arrowheads indicate uniform EdU labeled cells in the ventrolateral ectoderm. Panels b and c are left and right-side crops of ventral views of the trunk with anterior to left and posterior to the right. Asterisk denotes the mouth. The durations of sea-water incubation following EdU pulse is indicated on the right-hand corners. Scale bar: 50 μm

Another interesting observation from the pulse-chase experiments was that EdU labeling appeared homogeneous in some nuclei and stippled in other nuclei (e.g., Fig. 5). At 0–9 h after EdU pulse (stage 4 telotroch to stage 5 mid), the majority of EdU^+^ cells had a homogenous label (Fig. 4b, Fig. 5b, b’; Additional File 7: Fig. S4b–d). In contrast, 20 h after EdU pulse (stage 5 late), more EdU^+^ cells had stippled labeling (Fig. 4d, Fig. 5c, c’; Additional File 7: Fig. S4e, f, g, arrows). Furthermore, the pattern of EdU labeling (homogeneous versus stippled) was segregated based on distance from the ventral midline. Cells near the ventral midline exhibited weaker, stippled EdU labeling (Fig. 5c, c’; arrows), whereas cells positioned more laterally in the neuroectoderm had homogenous EdU labeling (Fig. 5b, b’, c, c′, closed arrowheads). Furthermore, 12–20 h after pulse, the weakly-labeled EdU^+^ cells were only detected near the surface of the neuroectoderm but not subsurface (Fig. 4c, c.1, c.2, Additional File 7: Fig. S4e, e.1, e.2, arrows), but by 36–48 h after pulse, some were also visible in more basal layers of the neuroectoderm (Fig. 4d, d.1, d.2, Additional File 7: Fig. S4f, f.1, f.2; open arrowheads). Based on the patterns we observed, the weakly-labeled, stippled EdU^+^ cells near the midline at later stages could be actively proliferating NPCs that gradually lost the EdU label with each round of mitosis, similar to previous observarions in the annelid *Platynereis dumerilii* [65]. In contrast, the few stippled EdU cells that were detected at time zero (Fig. 5b, arrows) could be cells that only underwent a partial S-phase during the EdU pulse, resulting in an incomplete labeling of the chromatin. As there were only a small number of stippled cells at time zero, we do not think they greatly contributed to the later population of stippled cells. Homogenously labeled EdU^+^ cells detected at later timepoints (20–72 h) may represent cells that exited the cell-cycle soon after EdU incorporation.

**Fig. 6:**
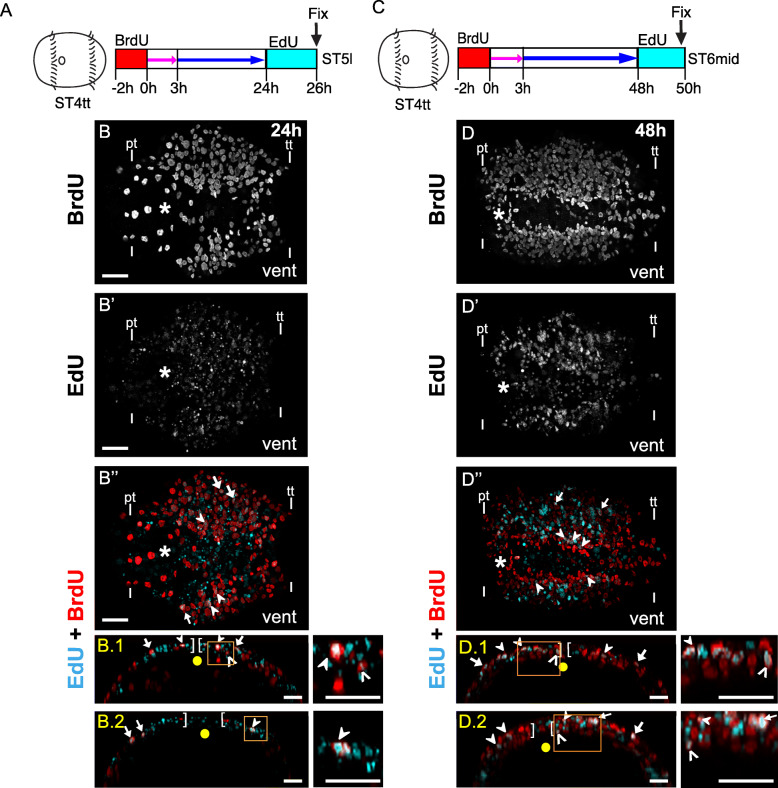
Actively dividing NPCs are localized more ventrally near the midline. **a, c** Schematic showing stage 4 telotroch animals exposed to BrdU (red) for 2 h followed by a 3 h thymidine chase (pink arrow) and sea-water incubation (blue arrow) until 24 h **a** and 48 h **c** respectively before a 2 h EdU (cyan) pulse. **b, b′, b″, b.1, b.2, d, d′, d″, d.1, d.2** Confocal micrographs of BrdU pulse-chase-wait-EdU experiments showing BrdU (red) and EdU (cyan) after chase for 24 h **b–b″, b.1, b.2** and 48 h **d–d″, d.1, d.2**. Panels **b, b′, b″, d, d′, d″** represent ventral views of the trunk neuroectoderm, with anterior to the left and posterior to the right, showing single BrdU^+^**b, d**, EdU^+^**b′, d″** and EdU/BrdU dual-labeled cells **b″, d″**. Panels b.1, b.2, d.1, d.2 show orthogonal sections through the ventral trunk neuroectoderm of **b**″ and **d**″ respectively. b.1 and d.1 are transverse sections through the anterior trunk neuroectoderm, while b.2 and d.2 are through the posterior trunk neuroectoderm. In b.1, b.2, d.1, and d.2, orange boxes indicate regions that have been cropped and enlarged 3 times in the respective adjacent panels for better visualization of EdU/BrdU dual-labeled cells. In b", d", b.1, b.2, d.1, d.2, closed arrowheads indicate surface EdU^+^/BrdU^+^ dual labeled cells in the trunk neuroectoderm while open arrowheads indicate subsurface dual labeling. Arrows point to the ventrolaterally located surface EdU^+^/BrdU^+^ dual labeled cells. Asterisk denotes the position of the mouth. In all orthogonal views (b.1, b.2, d.1, d.2), apical is up and basal is down and the yellow dot denotes the ventral midline. Square brackets indicate the boundaries between the neuroectoderm and neurotroch. Prototroch (pt) and telotroch (tt) are indicated by dashes. The channel visualized is indicated on the left of each figure panel. ST4tt: Stage 4 telotroch, ST5l: Stage 5 late, ST6mid: stage 6 mid. Vent, ventral. Scale bar in panels **b–d**″: 25 μm. Scale bar in panels b.1, b.2, d.1, d.2: 15 μm

To test for the presence of dedicated NPCs, i.e., cells that divide multiple times across neurogenesis, we performed BrdU pulse-chase-wait-EdU sequential labeling. Stage 4 telotroch larvae were exposed to BrdU for 2 h, chased with thymidine for 3 h, allowed to develop until 24 or 48 h in seawater (stage 5 and 6, respectively), incubated in EdU for 2 h, and then fixed (Fig. 6a, c). At both timepoints, BrdU^+^/EdU^−^ cells, derived from cells initially labeled with BrdU, were present throughout the neuroectoderm — from ventral to lateral and apical to basal (Fig. 6b–b.2, d–d.2). In addition to the BrdU^+^/EdU^−^ cells, we also observed BrdU^+^/EdU^+^ cells at both timepoints, many of which were localized near the ventral midline in the neuroectoderm (Fig. 6b–d; closed and open arrowheads), further confirming that these cells may be dedicated NPCs. In the 24 h experiment, EdU^+^/BrdU^+^ cells were primarily observed in surface populations of the trunk (Fig. 6b, b’, b″, b.1, b.2; closed arrowheads and arrows) with only a few being subsurface (Fig. 6b.1; open arrowheads). In the 48 h experiment, the BrdU^+^/EdU^+^ cells near the ventral midline were in both surface (Fig. 6d, d’, d”, d.1, d.2; closed arrowheads) and subsurface populations (Fig. 6d, d’, d”, d.1, d.2; open arrowheads); surface dual-labeled cells were more common than subsurface ones. The few BrdU^+^/EdU^+^ cells in the ventrolateral neuroectoderm were apically-localized (Fig. 6b”, b.1, b.2, d”, d.1, d.2, arrows).

### SoxB and proneural homologs are expressed in proliferating NPCs during CNS development

**Fig. 7:**
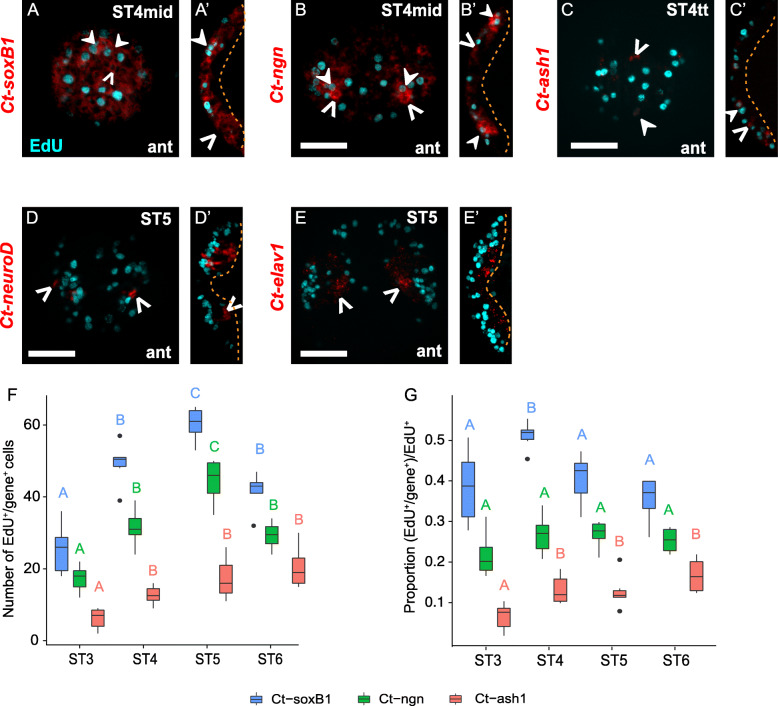
Gene expression in head EdU^+^ cells. **a–e**’ A 30-min EdU pulse (cyan) at different stages of neurogenesis was combined with FISH (red). **f, g** The number **f** and proportion **g** of EdU^+^ cells expressing a specific gene (colored boxes) in the brain were scored and plotted at different developmental stages. Anterior **a, b, c, d, e** and ventral **a’, b′, c′, d’, e’** views of the anterior ectoderm are shown. Overlap of EdU^+^ cells with *Ct-soxB1* (A, A’), *Ct-ngn* (B, B′), *Ct-ash1***c, c′**, *Ct-neuroD***d, d**’, and *Ct-elav1***e, e**’ are shown. In panels A–E’, closed arrowheads indicate EdU^+^/gene^+^ cells while open arrowheads show EdU^−^/gene^+^ cells. Dashed orange lines in **a’, b′, c′, d**’, and **e**’, demarcate the basal boundary of the brain. In **f, g**, capital letters above the boxplots indicate statistical groups comparing cell counts or proportions at different stages based on ANOVA analysis. Boxplots with the same letter are not significantly different. Those with different letters are significantly different, with *p* < 0.05 after correction for multiple comparisons. Upper and lower bounds of the box plot indicate the 3rd and 1st quartiles while the middle line inside the boxplot indicates the median. The ends of the whiskers represent the 5th and 95th percentiles, black dots represent outliers (±3 S.D.). ant, anterior; vent, ventral. ST4mid: Stage 4 middle, ST4tt: Stage 4 telotroch, ST5: Stage 5, ST6: Stage 6. Scale bar: 50 μm

**Fig. 8:**
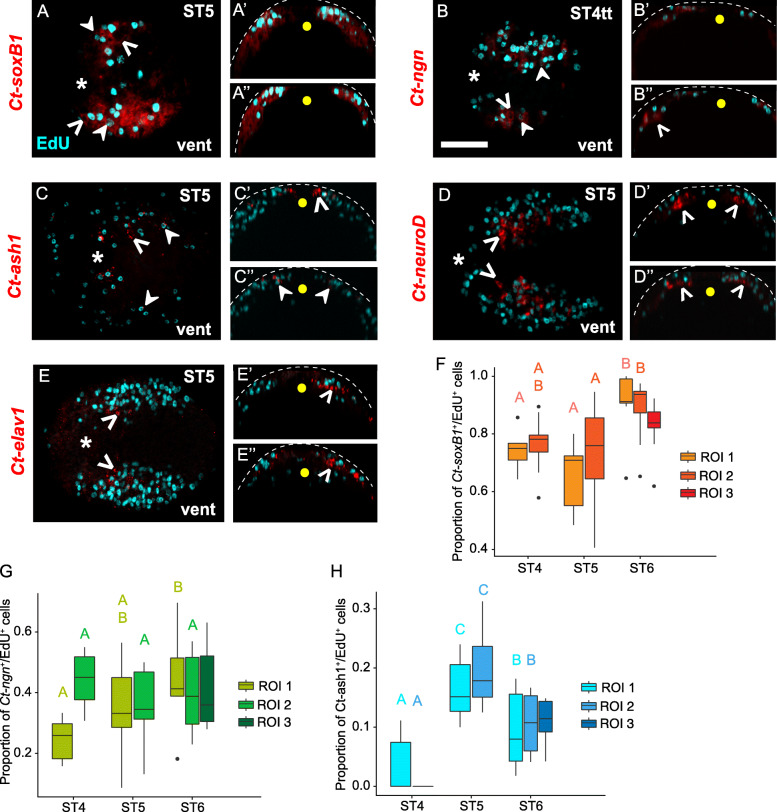
Gene expression in trunk EdU^+^ cells. **a–e**″ 30-min EdU pulse (cyan) at different stages of neurogenesis was combined with FISH (red). Ventral views of the trunk neuroectoderm **a, b, c, d, e** and transverse sections through the anterior **a’, b′, c′, d′, e**′, and posterior **a″, b″, c″, d″, e**″ trunk neuroectoderm are shown. Overlap of EdU^+^ cells with *Ct-soxB1***a–a**″, *Ct-ngn***b–b**″, *Ct-ash1***c–c**″, *Ct-neuroD***d–d**″, and *Ct-elav1***e–e**″ are shown. In panels **a–e**″, closed arrowheads indicate EdU^+^/gene^+^ cells while open arrowheads show EdU^−^/gene^+^ cells. In ventral view panels **a–e**, orientation of the animal is indicated in the bottom right corner, and developmental stage is indicated in the top right corner. **f–h** The proportion of EdU^+^ cells expressing a *Ct-soxB1* (F), *Ct-ngn***g**, and *Ct-ash1***h** in segments 2–4, 5–7, and 8–10 in the VNC were scored and plotted at different developmental stages. In **f**–**h**, capital letters on top of the boxplots (e.g. A, B etc.) indicate statistical significance computed using mixed effects model analysis. The upper and lower bounds of the box plot indicate the 3rd and 1st quartiles while the middle line inside the boxplot indicates the median. The ends of the whiskers represent the 5th and 95th percentiles. Black dots represent outliers (±3 S.D.). In orthogonal views, apical is up, basal is down and yellow dot shows the position of the ventral midline. White dotted line marks the apical boundaries of the neuroectoderm. In ventral views, anterior is to the left. An asterisk denotes the mouth. Ant, anterior; vent, ventral. ST4mid: Stage 4 middle, ST4tt: Stage 4 telotroch, ST5: Stage 5, ST6: Stage 6. Scale bar: 50 μm

To better understand which genes are expressed in dividing NPCs, we combined 30-min EdU pulses with FISH for *Ct-soxB1*, *Ct-neurogenin* (*Ct-ngn*), *Ct-achaete-scute homolog 1* (*Ct-ash1*), *Ct-neuroD*, and *Ct-elav1* across different stages of neurogenesis (Figs. 7, 8, Additional File 10: Fig. S5). Out of the five genes investigated, only *Ct-soxB1*, *Ct-ngn,* and *Ct-ash1* were found to be expressed in EdU^+^ cells. Previous work determined that *Ct-soxB1* and *Ct-ngn* are expressed at the onset of neurogenesis — in the anterior neuroectoderm at stage 3 and in the trunk neuroectoderm at stage 4 [67]. We observed *Ct-soxB1* expression in EdU^+^ and EdU^−^ cells in the surface and subsurface in the anterior and trunk neuroectoderm from stages 4–6 (Fig. 7a, a’, Fig. 8a–a”, Additional File 10: Fig. S5a–a”). In contrast, *Ct-ngn* was expressed exclusively in apically-localized cells in the anterior and trunk neuroectoderm at stage 4 (Fig. 7b, b’, Fig. 8b-b”), and in both surface and subsurface cells at stages 5 and 6 (Additional File 10: Fig. S5b–b″; data not shown). In the head, the subsurface expression of *Ct-ngn* at these later stages did not overlap with EdU (data not shown), whereas in the trunk, the subsurface expression of *Ct-ngn* did overlap with EdU (Additional File 10: Fig. S5b–b”).

We also quantified the number and proportion of EdU^+^ cells that expressed *Ct-soxB1*, *Ct-ngn*, and *Ct-ash1* in the anterior neuroectoderm (Fig. 7f, g) and the trunk neuroectoderm (Fig. 8f-h; Additional File 10: Fig. S5f–h). To statistically compare the number and proportion of EdU^+^ cells expressing these genes in the anterior neuroectoderm at stages 3–6, we performed an ANOVA for each gene. In the head, the total number of EdU^+^/*Ct-soxB1*^+^ (ANOVA, F_3,19_ = 37.05, *p* < 0.001) and EdU^+^/*Ct-ngn*^+^ (ANOVA, F_3,18_ = 30.43, *p* < 0.001) cells progressively increased until stage 5 and then declined by stage 6 (Fig. 7f, Additional File 8: Table S5). The proportion of EdU^+^ cells expressing *Ct-soxB1* in the anterior neuroectoderm peaked at stage 4 (ST4, 0.524 ± 0.013 S.E.M.) and was similar at stages 3, 5, and 6 (ANOVA, F_3,19_ = 6.516, *p* = 0.003). The proportion of EdU^+^ cells expressing *Ct-ngn* in the anterior ectoderm remained constant across stages 3–6 (ANOVA, F_3,19_ = 1.816, *p* = 0.180; Fig. 7G, Additional File 8: Table S5). In the trunk neuroectoderm, quantification was carried out across the same ROIs 1–3 as previously (Additional File 7: Fig. S4h) and at different time points (Fig. 8f-h, Additional File 10: Fig. S5f–h, Additional File 8: Table S6). To test for differences in the number and proportion of EdU^+^ cells expressing these genes in the trunk, we performed mixed effects modeling analysis for each gene (Additional File 8: Table S3, S4). These mixed effects models explained 42 to 87% of variation in co-expression counts and proportions (Additional File 8: Table S3). The total number of EdU^+^/*Ct-soxB1*^+^ cells in ROI 1 and 2 generally increased from stages 4–6 (Additional File 10: Fig. S5f, Additional File 8: Table S6). The number of EdU^+^/*Ct-ngn*^+^ cells in ROI 1 increased from stage 4 to 5 and then remained constant, whereas, the number of EdU^+^/*Ct-ngn*^+^ cells in ROI 2 remained constant from stage 4 to 5 and then increased (Additional File 10: Fig. S5g, Additional File 8: Table S6). The proportion of EdU^+^/*Ct-soxB1*^+^ and EdU^+^/*Ct-ngn*^+^ cells in ROI 1 and 2 was similar across stages, remaining constant from stage 4 to 5 for both and only increasing by stage 6 for EdU^+^/*Ct-soxB1*^+^ (Fig. 8f, g, Additional File 8: Table S6). At stage 6, ROI 3 had a higher number and a lower proportion of EdU^+^/*Ct-soxB1*^+^ cells relative to ROI 1 and 2 at the same stage (Fig. 8f, Additional File 10: Fig. S5f, Additional File 8: Table S6). In contrast, the number of EdU^+^/*Ct-ngn*^+^ cells in ROI 3 at stage 6 was similar to ROI 2 and possibly higher than ROI 1, while the proportion of EdU^+^/*Ct-ngn*^+^ cells across all three ROIs appeared similar (Fig. 8g, Additional File 10: Fig. S5g, Additional File 8: Table S6).

We previously showed that *Ct-ash1* is expressed at the onset of neurogenesis in the anterior neuroectoderm at stage 3 and slightly later in the anterior-most segments of the trunk neuroectoderm at stage 5 [66, 67]. At stage 3 in the anterior neuroectoderm, *Ct-ash1* is expressed in a small patch of cells, representing ~ 8% of EdU^+^ cells (Fig. 7f, g). At stages 4 and 5 in the head, *Ct-ash1* was largely expressed in apically-localized EdU^+^ and EdU^−^ cells; however, a few subsurface *Ct-ash1*^+^/EdU^−^ neuroectodermal cells were detected by late stage 4 and at stage 5 (Fig. 7c, c’ closed and open arrowheads). Interestingly, most *Ct-ash1*^*+*^ cells were found adjacent to EdU^+^ cells (e.g., Fig. 7c’; open arrowhead), indicating that *Ct-ash1* may be expressed in daughters generated by dividing NPCs. By stage 6, *Ct-ash1* was primarily localized to the lateral edges of the developing brain, where continued cell internalization is thought to be occurring [66, 67]. The number (ANOVA, F_3,19_ = 10.64, *p* < 0.001) and proportion (ANOVA, F_3,19_ = 6.675, *p* = 0.002) of EdU^+^/*Ct-ash1*^+^ cells increased from stage 3 to 4 and then remained fairly constant through stage 6 (Fig. 7f, g; Additional File 8: Table S5).

In the trunk neuroectoderm at stage 4, *Ct-ash1* was detected only in 1–2 cells in the trunk just posterior to the mouth [67]. Starting at stage 5, *Ct-ash1* was expressed in patches of apically-localized cells in segments 1–5 (Fig. 8c), encompassing ROI 1 and part of ROI 2. We scored expression as mentioned previously, and at stage 5 we found overlap with both EdU^+^ and EdU^−^ cells in ROI 1 and 2 (Fig. 8c–c”, closed and open arrowheads, respectively). By stage 6, *Ct-ash1* was expressed in both apical and basal cells in the trunk neuroectoderm and had expanded posteriorly to encompass ROI 3 (Additional File 10: Fig. S5c–c″, h). At mid-stage 6, we found *Ct-ash1*^+^/EdU^−^ cells near the ventral midline in ROI 1 (Additional File 10: Fig. S5c c’, open arrowheads), and *Ct-ash1*^+^/EdU^+^ cells near the ventral midline in ROI 2 and ROI 3 (Additional File 10: Fig. S5c, c″, closed arrowheads). By late stage 6, *Ct-ash1* expression was down-regulated from anterior to posterior until there was only expression in the posterior growth zone at stage 7 (previously reported in Sur et al. 2017). The number of *Ct-ash1*^+^/EdU^+^ cells in ROI 1 declined from stage 5 to 6 while the number of *Ct-ash1*^+^/EdU^+^ cells in ROI 2 remained unchanged from stage 5 to 6 (Additional File 10: Fig. S5h, Additional File 8: Table S3, S4, S6). In contrast the proportion of *Ct-ash1*^+^/EdU^+^ cells in both ROI 1 and 2 declined from stage 5 to 6 (Fig. 8h, Additional File 8: Table S6). In ROI 3, the number and proportion of *Ct-ash1*^+^/EdU^+^ cells were similar to that of ROI 2 at stage 6 (Fig. 8h, Additional File 10: Fig. S5h, Additional File 8: Table S6).

*Ct-neuroD* and *Ct-elav1* were detected in the anterior ectoderm beginning at stage 3 and stage 4, respectively, and in the developing ganglia of the presumptive VNC by stage 5 (Fig. 8d–d”, e–e”) [66, 67]. In both the head and trunk at all stages investigated, *Ct-neuroD* and *Ct-elav1* were expressed in basally-localized EdU^−^ cells and were completely excluded from EdU^+^ cells (Fig. 7d–e’, Fig. 8d–e”, Additional File 10: Fig. S5d–e”, open arrowheads). We did detect a few *Ct-neuroD*^+^/EdU^−^ cells near the apical surface in the brain at stage 5 (Fig. 7d’). In the trunk at stage 5, *Ct-neuroD* and *Ct-elav1* were expressed in developing ganglia in segments 2–4 and 5–6 (Fig. 8d, e). From stage 5 to mid-stage 6, *Ct-neuroD* and *Ct-elav1* expression expanded posteriorly throughout the ganglia of the VNC from anterior to posterior [67]. Then by stage 7, the number of EdU^+^ cells declined from the trunk neuroectoderm with the majority of EdU^+^ cells confined to the posterior growth zone (Additional File 10: Fig. S5a, e) and only 1–2 EdU^+^ cells remaining per segment (Additional File 10: Fig. S5d, e). *Ct-neuroD* was expressed only in the posterior few segments including the posterior growth zone, but not in EdU^+^ cells (Additional File 10: Fig. S5d–d”) while *Ct-elav1* was expressed strongly in all VNC ganglia (Additional File 10: Fig. S5e–e”).

### Co-expression of neurogenic homologs suggests a possible gene regulatory network

**Fig. 9:**
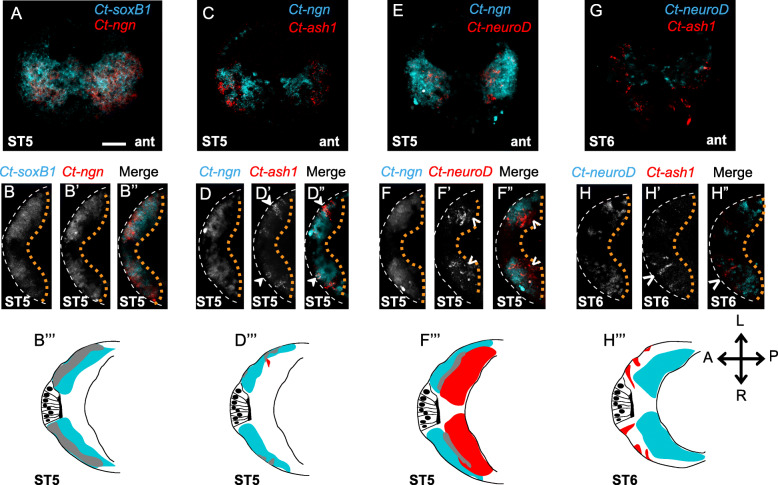
Spatial localization of different neurogenic homologs in the anterior neuroectoderm. Confocal images of double FISH are shown as anterior **a, c, e** and **g** and ventral (anterior to the left) views of the brain **b, d, f** and **h**. The genes used in double FISH are listed in the figure panels. **a–b***Ct-ngn* (red) expression overlaps a subset of the *Ct-soxB1*^+^ cells (cyan) in the head. **c–d***Ct-ngn* (cyan) and *Ct-ash1* (red) expression overlaps in a few cells in the head. **e–f***Ct-neuroD* (red) expression overlaps *Ct-ngn* (cyan) only in the intermediate brain. **g–h** No overlap detected between *Ct-ash1* and *Ct-neuroD*. Closed arrowheads indicate co-expression of two neurogenic homologs while open arrowheads indicate non-overlapping expression of neurogenic homologs in respective panels. Panels b''', d''', f''', h''' are diagrams representing co-expression of respective genes (gray) associated with figure panels b, d, f and h. The orange dotted lines indicate the basal edge of the brain while the white dashed lines indicate the apical boundary of the brain. The different developmental stages investigated are indicated at the lower left corner of each figure panel. Ant, anterior. Scale bar: 25 μm

**Fig. 10:**
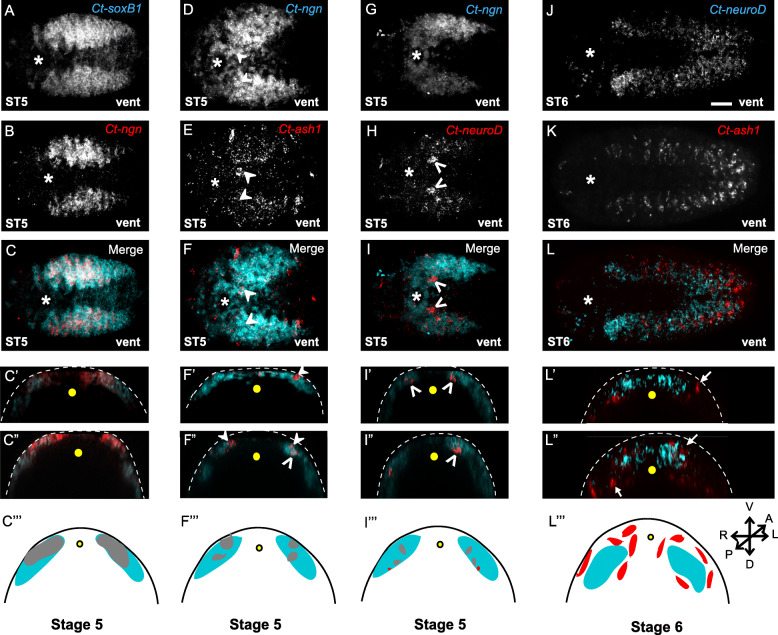
Co-expression of neurogenic homologs in the trunk neuroectoderm. **a–c** a subset of *Ct-soxB1*^+^ cells (cyan) which express *Ct-ngn* (red) along the more ventral regions. **d–f***Ct-ngn* (cyan) is expressed over a broad domain in the trunk ectoderm while *Ct-ash1* (red) is expressed in a punctate manner throughout the trunk ectoderm overlapping *Ct-ngn*^*+*^ cells. **g–i***Ct-neuroD* (red) overlaps some *Ct-ngn*^+^ cells (cyan) in the trunk neuroectoderm although some *Ct-neuroD*^+^/*Ct-ngn*^*−*^ can also be detected. (**j**–**l**) *Ct-neuroD* (cyan) and *Ct-ash1* (red) are expressed in non-overlapping domains in the trunk. **c', c", f', f", i', i'', l', l'**' are orthogonal views through anterior **c′,f′, i′, l**’ and posterior **c″, f″, i″, l**” segments of the trunk neuroectoderm shown in **c, f, i** and **l** respectively. The asterisk indicates the position of the mouth. **c″’, f″’, i″’, l**”’ are diagrams representing the orthogonal sections through the trunk neuroectoderm where colored regions represent relative co-expression (gray) or separate expression (cyan/red) of respective genes associated with figure panels **c, f, i** and **l**. In all figure panels, closed arrowheads indicate co-expression of two neurogenic homologs in surface cells while open arrowheads indicate co-expression in subsurface cells in respective panels. Arrows indicate non-overlapping expression of neurogenic homologs in panels **l’, l**”. Orientation of images are indicated on the lower right corner. In all orthogonal views apical is up and the yellow dot denotes the ventral midline. Dashed line indicates the apical edge of the transverse sections. The different developmental stages investigated are indicated at the lower left corner of each figure panel. Vent, ventral. Scale bar: 25 μm

Overall, the pattern of gene expression relative to EdU labeling suggests that proliferative NPCs in the head and trunk express *Ct-soxB1* and *Ct-ngn*, differentiating neural cells express *Ct-neuroD* and *Ct-elav1*, and cells transitioning between the two states may express *Ct-ash1*. To begin to generate a neurogenic GRN underlying brain and VNC development in *C. teleta*, we examined co-expression of *Ct-soxB1*, *Ct-ngn*, *Ct-ash1*, and *Ct-neuroD* at stages 4–6 using double FISH (Figs. 9 and 10; Additional File 11: Fig. S6, and data not shown). A large number of *Ct-soxB1*^+^ cells in both the anterior and trunk neuroectoderm co-expressed *Ct-ngn* at stages 4–6 (Fig. 9a–b; Fig. 10a–c; Additional File 11: Fig. S6a–c). At stage 4, *Ct-soxB1* and *Ct-ngn* overlapped apically in the anterior neuroectoderm (data not shown). At stages 5 and 6, *Ct-soxB1*^*+*^/*Ct-ngn*^+^ cells were localized to the surface and intermediate (i.e., just below the surface) layers of the anterior neuroectoderm (Fig. 9a–b”’, data not shown). In the trunk at stages 4–6, *Ct-soxB1*^+^/*Ct-ngn*^+^ cells were found throughout the neuroectoderm (Fig. 10a–c”, Additional File 11: Fig. S6a–c″) in both surface and subsurface cells (Fig. 10c’, c", Additional File 11: Fig. S6c’, c″, data not shown). Across all stages examined, all *Ct-ngn*^+^ cells were also found to express *Ct-soxB1*.

At stages 5 and early 6, we also observed *Ct-ngn*^+^/*Ct-ash1*^+^ (Fig. 9c–d”’; Fig. 10d–f”’, Additional File 11: Fig. S6d–f″) and a few *Ct-ngn*^+^/*Ct-neuroD*^+^ (Fig. 9e-f”’; Fig. 10g–i”’) cells in the anterior and trunk neuroectoderm. In contrast, we never observed *Ct-ash1*^+^/*Ct-neuroD*^+^ (Fig. 9g–h”’; Fig. 10j–l”’) or *Ct-ash1*^+^/*Ct-elav1*^+^ (Additional File 11: Fig. S6g–i″) cells in the head or trunk during this time. At stage 5 and early 6, a majority of *Ct-ash1*^+^ cells, both surface and subsurface, also expressed *Ct-ngn* in the anterior and trunk neuroectoderm (Fig. 9d–d”’, closed arrowheads; Fig. 10d–f”’, closed and open arrowheads; Additional File 11: Fig. S6d–f″, closed arrowheads). Furthermore, two *Ct-ash1*^+^/*Ct-ngn*^+^ cells were reproducibly detected in the anterior-most ganglion of the VNC, just posterior to the mouth (Fig. 10 d–f, closed arrowheads). A small number of *Ct-ngn*^+^/*Ct-neuroD*^+^ cells were found to be localized just below the surface, to the intermediate region of the anterior neuroectoderm at stage 5 (Fig. 9e–f) and just below the surface in the anterior trunk neuroectoderm and at the surface in the posterior trunk neuroectoderm at stage 5 (Fig. 10g–i”’, open arrowheads). By late stage 6, expression of both *Ct-ash1* and *Ct-ngn* began to be downregulated in the ganglia of the VNC in an anterior to posterior wave as *Ct-elav1* expression turned on. After stage 6, expression of *Ct-ash1* and *Ct-ngn* was no longer detectable in the VNC, and was instead localized to the posterior growth zone, from which new ganglia of the VNC are added (Additional File 11: Fig. S6g–i) [67].

## Discussion

### Model of brain neurogenesis in *C. teleta*

**Fig. 11:**
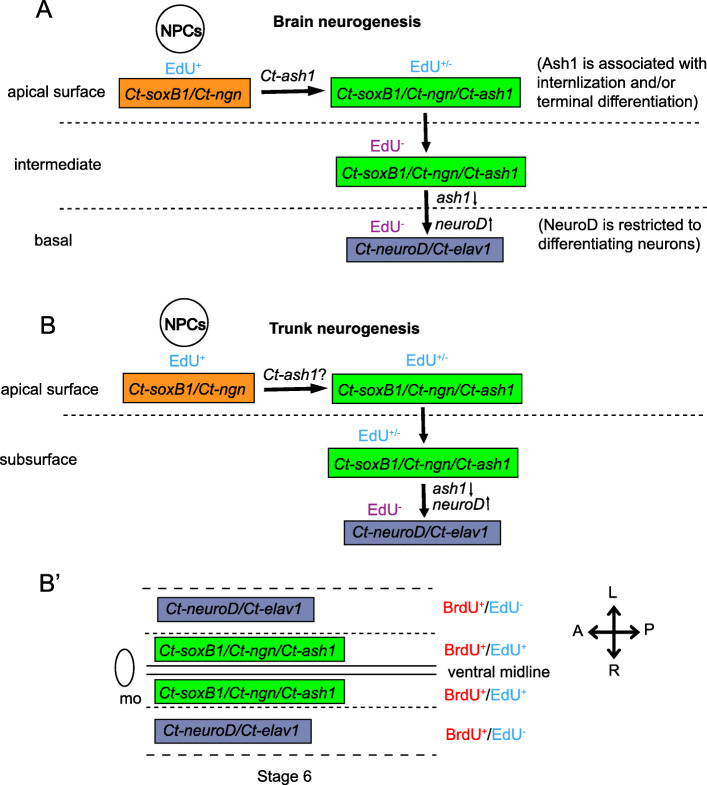
Models of *C. teleta* brain and trunk neurogenesis. **a** A hypothetical GRN underlying brain neurogenesis. There is a transition from apical proliferative EdU^+^ cells to more basal differentiating neural cells that are spatially segregated from each other. Different combinations of transcription factors are expressed along these different spatial domains in the head as indicated. **b, b**′ A hypothetical GRN underlying VNC neurogenesis showing different EdU labeled cell populations with differential expression of transcription factors in the surface versus subsurface cells in the trunk neuroectoderm along the apical-basal axis **b** and along the dorsoventral axis **b**′. fg, foregut; A: anterior; P: posterior; D: dorsal; V: ventral. Dashed lines indicate the demarcations between the different spatial domains

Our results, combined with previous work [66, 67], allowed us to generate a model of brain neurogenesis in *C. teleta* (Fig. 11a). The anterior neuroectoderm at stage 3 is a simple epithelium comprising many *Ct-soxB1*^+^/EdU^+^ cells, which could represent both neural and epidermal precursors. A subset of these cells also express *Ct-ngn*, and these cells may be the neural precursor or stem cells (NPCs). As neurogenesis proceeds in the head (stages 4–6), NPCs divide apically and some daughters begin to express *Ct-ash1*, which may trigger ingression. This hypothesis is based on the observation that a much smaller percentage of apical EdU^+^ cells express *Ct-ash1* (e.g., ~ 14% at stage 5) versus *Ct-ngn* (e.g., ~ 28% at stage 5) or *Ct-soxB1* (e.g., ~ 42% at stage 5). Most *Ct-ash1*^+^ cells, both apical and intermediate (i.e., just below the surface), also appeared to co-express *Ct-ngn*, but not the converse. Finally, *Ct-ash1* was often expressed in apical EdU^−^ cells that were adjacent to EdU^+^ cells as well as in a small number of internalized EdU^−^ cells.

After ingression, we suggest that cells begin differentiating, first expressing *Ct-neuroD* and then *Ct-elav1*, to form the brain. Interestingly, we observed a small number of *Ct-ngn*^+^/*Ct-neuroD*^+^ cells at stages 5 and 6 that were positioned just below the apical surface (i.e. intermediately-localized); we did not observe any *Ct-ngn*^+^/*Ct-elav1*^+^ cells at these same stages. This suggests that *Ct-ngn*, in addition to a possible function in NPCs, may also have a transient role in neural cells just prior to their differentiation, as *Ct-neuroD* did not overlap with EdU. On the other hand, *Ct-ash1* was not seen to overlap with either *Ct-neuroD* or *Ct-elav1*. This suggests that *Ct-ash1* may act as a molecular switch between *Ct-soxB1*/*Ct-ngn*-mediated NPC proliferation and *Ct-neuroD*/*Ct-elav1*-mediated differentiation. In this case *Ct-ash1* would be downregulated just prior to the onset of differentiation. Another interpretation is that different neural subtypes arise based on the combination of bHLH transcription factors expressed, e.g. *Ct-ngn*^+^/*Ct-neuroD*^+^, *Ct-ngn*^+^/*Ct-ash1*^+^, or *Ct-ash1* alone. A similar scenario is observed in vertebrates (mice, chicken, zebrafish and *Xenopus laevis*) where Achaete-scute homologs (Mash1, Cash1, Zash1a, Xash1) are expressed in a subpopulation of cells, driving them to differentiate as GABAergic neurons. In contrast Neurogenin/NeuroD^+^ cell populations give rise to glutaminergic neurons [8, 22, 74–84]. However, the early onset of *Ct-ngn* and *Ct-ash1* expression in *C. teleta*, prior to any signs of ingression or differentiation, and expression in EdU^+^ cells suggests that these genes have an earlier function in neurogenesis. Other hypotheses are also plausible and await functional analysis of individual genes for further testing.

Throughout brain neurogenesis, an apical repertoire of proliferative NPCs is maintained. The number of apically-localized EdU^+^ labeled at stage 4 remained fairly constant through stage 6 (48 h later) as assayed by EdU pulse-chase while the number of basally-localized EdU^+^ cells increased across the same timeframe. EdU/PH3 and BrdU pulse-chase-wait-EdU labeling further confirm this and suggest that internalized cells do not undergo mitosis. However, it is also possible that internalized cells could undergo one last terminal cell division or could be dividing at a much slower rate than apical NPCs. We favor the previous hypothesis because most, if not all, basally-localized cells express *Ct-neuroD* and *Ct-elav1*, which have been shown to function post-mitotically in cnidarians, insects and chordates [85–90]. These data support a spatial segregation between apically-dividing NPCs and basal, post-mitotic neural cells in the anterior neuroectoderm.

### Model of VNC neurogenesis in *C. teleta*

We found many similarities between neurogenesis in the anterior and trunk neuroectoderm, but also a few differences. Our model for VNC neurogenesis in *C. teleta* (Fig. 11b, b’) starts at early stage 4, when the trunk neuroectoderm is composed of proliferative NPCs that express *Ct-soxB1* and *Ct-ngn*. One notable difference from the brain is that *Ct-ash1* and *Ct-neuroD* expression initiates later (early stage 5) than *Ct-ngn* (early stage 4) in trunk neuroectoderm versus at approximately the same time (early stage 3) in the anterior neuroectoderm (Fig. 11b). By late stage 4, basally-localized nuclei in the neuroectoderm begin to be visible on either side of the ventral midline in the anterior-most segments, possibly representing the first internalized cells. Beginning at stage 6, there appear to be fewer EdU^+^ cells than at stage 5 in the first three anterior segments, coincident with strong *Ct-ash1*, *Ct-neuroD*, and *Ct-elav1* expression. The patterns of gene co-expression and EdU/gene overlap at stages 5 and early 6 in the trunk appear to be largely the same between the brain and VNC with *Ct-ngn* and *Ct-ash1* expressed in apical EdU^+^ cells and in intermediate EdU^+^ and EdU^−^ cells. A few intermediately-localized *Ct-ngn*/EdU^−^ cells co-expressed *Ct-neuroD* similar to the brain at stages 5 and 6. This highlights one difference, which is that a small number of subsurface EdU^+^ cells were observed in the developing VNC, which is further discussed below. We did not observe expression of *Ct-ash1* in *Ct-neuroD*^+^ or *Ct-elav1*^+^ cells, similar to our findings in the brain.

Other differences between the anterior and trunk neurectoderm were the patterns of dividing cells. First, as mentioned previously, we observed dividing EdU^+^ and PH3^+^ subsurface cells in the trunk neuroectoderm. We also found subsurface EdU^+^/BrdU^+^ cells after BrdU pulse-chase-wait-EdU experiments, although at a much lower frequency than apical EdU^+^/BrdU^+^ cells, suggesting that subsurface cells may be cycling at a slower rate or may undergo fewer divisions relative to surface NPCs. Secondly, along the medial-lateral axis in the trunk neuroectoderm, actively dividing NPCs appear to be localized near the ventral midline. In support of this, in posterior segments at stage 5, we observed “stippled” EdU labeling of cells following EdU pulse-chase experiments as well as EdU^+^/BrdU^+^ cells following BrdU pulse-chase-wait-EdU experiments, both indicative of actively dividing NPCs, localized near the ventral midline. In contrast, we observed more “uniform” EdU labeling of cells in lateral regions of the trunk neuroectoderm, suggesting that these cells may be less proliferative relative to cells closer to the ventral midline. Similar patterns of uniform and stippled labeling were observed after EdU pulse-chase experiments in *P. dumerilii* [65]. Furthermore, the “stippled” EdU^+^ nuclei following EdU pulse-chase assays were detected primarily near the apical surface of the trunk neuroectoderm. Taken together, these data suggest that actively-dividing NPCs may be localized closer to the ventral midline, in the apical surface of the trunk neuroectoderm. In contrast to the brain, there also appears to be a subset of dividing cells, possibly progenitor cells with a reduced proliferative potential, more laterally and below the surface in the trunk neuroectoderm. EdU pulse-chase experiments conducted in the errant annelid *P. dumerilii* also revealed similar spatial boundaries between actively dividing NPCs and restricted progenitors in the trunk neuroectoderm [65]. Our data is in line with previous evidence from *C. teleta* that highlighted different mechanisms underlying neural fate specification for the brain and VNC [91].

Interestingly, EdU pulse-chase experiments initiated at stage 4 found labeled cells in the newly-formed, posterior segments at stage 6 and 7. Two possible scenarios are that 1) NPCs in the anterior trunk neuroectoderm that were labeled at stage 4 contributed to these posterior ganglia, possibly crossing segmental boundaries. Alternatively, 2) *C. teleta* may have posteriorly-localized ectodermal teloblasts or naïve, dividing ectodermal cells that were labeled at stage 4 and that then generated new NPCs in the posterior segments at later stages. In support of the second hypothesis, a posterior ring of *nanos*^+^ ectodermal cells was identified in *C. teleta*, just anterior to the telotroch at stage 4 [92].

### Interpreting developmental differences between the brain and VNC in *C. teleta*

Differences in developmental mechanisms in the brain and VNC of *C. teleta* (this paper and [91]) can be interpreted in multiple ways. We previously hypothesized [91] that the last common ancestor of annelids, or possibly annelids and mollusks, had two separate developmental neural modules — an anterior neural system and a trunk neural system. This hypothesis was based on our finding that the brain (anterior neural system) in *C. teleta* appears to be specified autonomously while the VNC (trunk neural system) is conditionally specified by signals from vegetal macromeres. This hypothesis was further supported by fate mapping and blastomere isolation studies from a few other spiralian taxa. This led us to speculate that there may have been separate selection pressures on the two neural system modules (the anterior neural system being selected upon to be able to respond to environmental cues earlier in development), and therefore slightly different evolutionary trajectories for each.

Alternatively, or in parallel with our hypothesis of differing selection pressures, the brain develops earlier and in a different embryonic context than the VNC, which could lead to developmental differences. For example, the two tissues experience different signals within the anterior-posterior and dorsal-ventral patterning systems. Although the spatial segregation of NPCs is different in the brain and VNC of *C. teleta*, our findings, though not functional, suggest very similar neurogenic GRNs between the two systems. The one genetic difference we detected was the later onset of *Ct-ash1* and *Ct-neuroD* relative to other neurogenic genes in the trunk. This combined with the shared molecular signature with NPCs in other taxa (discussed below) suggests homology between brain and VNC NPCs, which is not surprising.

Uncoupling of head and trunk development can be found in many taxa, especially in indirect developing larvae (e.g. indirect developing echinoids [93], hemichordates [94] and nemerteans [95].) For example, Gonzalez et al. (2017) found differences in when and where expression of anterior and posterior *hox* genes initiated in the tornaria larva and juvenile of the indirect-developing hemichordate *Schizocardium califoricum* and the embryo and juvenile of the direct-developing hemichordate *Saccoglossus kowalevskii* [94]. The indirect-developing tornaria larva has a delayed onset of trunk development (i.e. expression of posterior *hox* genes) and is essentially a “swimming head”. *C. teleta* also is an indirect developer, but we do not think this explains the presence of different developmental modes for the brain and VNC. Unpublished data (lineage tracing from embryo to juvenile and analysis of apoptosis in the CNS during and after metamorphosis, NPM) as well as published results [70, 91] have led us to think that the both the anterior and trunk (at least the first 13 segments of the VNC) adult nervous system are formed during larval development in *C. teleta*. We do see an anterior-to-posterior progression of CNS development in *C. teleta*, i.e. the brain begins development first, followed by the anterior-most ganglia in the VNC, etc. However, the timing between onset of neurogenesis in the anterior VNC (early stage 5) and the posterior VNC (early stage 6) in the larva overlaps with brain neurogenesis.

Finally, it is worth noting that Tosches and Arendt (2013) have proposed that the sensory-neurosecretory and the locomotory centers in the brain of extant bilaterians (insects, annelids, and vertebrates) were initially two spatially separate neural centers, one apical and one blastoporal during gastrulation. They hypothesized that these centers ‘fused’ in the last common ancestor of bilaterians [96]. Based on the developmental differences found in the brain and VNC of *C. teleta*, we propose that these two regions of the CNS are distinct modules, possibly with separate evolutionary pressures. This is a somewhat different scenario than Tosches and Arendt’s bilaterian chimeric brain hypothesis, since we propose that in *C. teleta* (an extant species) the VNC and the entire brain are separate developmental modules.

### Comparisons of *C. teleta* neurogenesis with other animals

To better understand nervous system development in annelids, we compared our findings with data from other animals. An apical-basal spatial segregation between NPCs and post-mitotic neural cells has been observed in many animals, suggesting that this may be an ancient feature of nervous system development. Furthermore, expression of *soxB* and bHLH gene homologs (*achaete-scute* and *neurogenin*) in the neuroectoderm is another common theme, although the regulation and function of the homologs may be different across taxa. Based on the traditional view in vertebrates and arthropods, SoxB1 protein homologs are known to maintain proliferative NPCs [5, 21, 29–34, 38, 97]. Achaete-Scute protein homologs induce specification of a neural fate in insects [22, 54, 98] and promote neural differentiation in vertebrates and non-insect arthropods [5, 10, 12, 14, 27, 99–103]. Recent genome-wide transcription factor binding studies in vertebrates and *D. melanogaster* have highlighted some less well-understood functions of these two groups of proteins — SoxB1 homologs can induce neural differentiation [38, 50] and the mouse Achaete-Scute homolog Ascl1 can promote cell proliferation [24, 27, 56, 57]. In mice, *sox1* was found to be expressed in postmitotic cells necessary for proper neuronal migration and subtype identity in the ventral telencephalon [46–49]. Mouse *sox2* was found to be present in some mature neurons and Sox2 knockdown resulted in a loss of GABAergic neurons, hippocampal malformation and impaired neuronal migration [46–49] while Sox3 was found to be required for the establishment of the hypothalamus-pituitary axis [46–49]. In *D. melanogaster*, SoxNeuro was found to be involved in neuronal differentiation and axonal patterning [38, 50]. On the other hand, mouse Ascl1 and insect Asense have also been found to directly upregulate cell-cycle progression genes thereby maintaining NPC proliferation [24, 27, 56, 57].

Similarly, in *C. teleta*, *Ct-soxB1* is expressed in dividing NPCs as well as in post-mitotic neural cells indicating a multifaceted function from NPC maintenance to terminal differentiation. One SoxB homolog has been identified in the annelid *P. dumerilii* (Errantia) that is expressed early during neurogenesis but never overlaps *Pdu-neurogenin* or *Pdu-achaete-scute* [62]. This fits the traditional vertebrate model but contrasts with our observations in *C. teleta* of co-expression of *Ct-soxB1* with *Ct-ngn* and likely *Ct-ash1*, although we were unable to verify the latter. Our data also show that *Ct-ngn* and *Ct-ash1* are expressed in dividing NPCs in both the head and trunk neuroectoderm of *C. teleta*, indicating a probable function in promoting NPC proliferation. Similarly, in *P. dumerilii*, apically-localized, proliferative NPCs expressing *Pdu-ngn* and *Pdu-ash* were spatially segregated from basally-localized, non-dividing neural cells expressing *Pdu-elav1* and *Pdu-syt* [63, 65]. In both *P. dumerilii* and *C. teleta*, the early onset and broad pattern of expression of *neurogenin* homologs in EdU^+^ cells in the neuroectoderm suggests that Ngn functions in proliferative NPCs [65] . In contrast Ash may drive cells to become internalized and to be less proliferative, given its expression in only a few EdU^+^ cells and its downregulation prior to neural differentiation. NeuroD homologs in *C. teleta* and *P. dumerilii* appear to have different functions. *Ct-neuroD* is exclusively expressed in EdU^−^, basal cells and overlaps with *Ct-elav1*, while *Pdu-neuroD* is expressed early throughout the trunk neuroectoderm in *P. dumerilii* [63, 67]. Unraveling such novel roles of these bHLH homologs in annelids concomitant with recent data from vertebrates and insects suggest that the roles of these transcription factors may be more nuanced that previously thought.

The annelid ancestral state representing apical-basal segregation between NPCs and neural cells has also been observed in sister spiralian taxa such as the mollusk *Aplysia californica*, where NPCs arise within a proliferative zone in the ectoderm and then their daughters migrate to the nearest ganglia individually or in groups where they differentiate [104]. Among platyhelminths such as the planarians *Schmidtea mediterranea* and *Schmidtea polychroa*, neurogenesis occurs from a proliferative progenitor population, a subset of which express *soxB1* homologs [105–107]. Similar to annelids, expression of *soxB1* homologs in the planarian *S. polychroa* is also consistent with a role in specification and maintenance of proliferative NPCs as well as in neural differentiation [106]. Achaete-scute homologs in the planarians *S. mediterranea* and *S. polychroa* were found to be involved in all aspects of neurogenesis such as stem cell maintenance, neural differentiation and neuronal subtype specification. One of the *ash* paralogs in each planarian was expressed overlapping mitotic cells similar to *C. teleta* and *P. dumerilii*; however, in both planarians, *neuroD* homologs were found to be expressed in early progenitors similar to *P. dumerilii* but not *C. teleta* [105, 106]. Additionally, in the arrow-worm *Spadella cephaloptera* (Gnathifera), which may be in a sister clade to Spiralia [108], NPCs in the ventral neuroectoderm are apically-localized and are spatially separated from basally-localized, differentiating neurons [109, 110]. However, the role of bHLH homologs during neurogenesis in these taxa has not been investigated yet, and this may serve as important information to deduce if NPCs expressing bHLH homologs are an ancestral feature of Spiralia.

In most other emerging study organisms, limited data indicate that Neurogenin and Achaete-Scute homologs function in differentiating neural cells, consistent with the traditional roles in well-studied insects and vertebrates. For example, in non-insect arthropods such as chelicerates and myriapods, Achaete-Scute homologs promote neural differentiation [10, 15, 101, 111]. Only one arthropod Neurogenin homolog has been identified in *D. melanogaster*, Tap, which functions in axonal growth and guidance [112–114]. Similarly, in non-chordate deuterostomes such as the sea-urchin *Lytechinus variegatus*, Ngn and Ash homologs specify distinct neuronal subtypes (cholinergic versus serotonergic), similar to vertebrates [115]. In the cnidarian *N. vectensis*, the bHLH homolog *NvAth-like*, which is phylogenetically related to *Ct-ngn*, is also expressed in dividing cells and never co-express the *N. vectensis ash* homolog, *Nv-AshA*. *Nv-ashA* was completely excluded from dividing cells [58], possibly demonstrating a role in neural differentiation, as seen in vertebrates. Therefore, a possible role of these bHLH factors in dividing NPCs may have evolved convergently in annelids or may have been an ancient feature in the last common ancestor of Bilateria, given our data from the annelid *C. teleta* and similar recent observations in vertebrates, insects and cnidarians.

## Conclusions

Our data elucidate cellular and molecular mechanisms underlying neurogenesis in the annelid *C. teleta*, thus promoting a better understanding of the evolution of neurogenesis. Our work reveals the dynamics of dividing NPCs and their daughters in the anterior and trunk neuroectoderm as well as important roles of neurogenic homologs in each aspect of neurogenesis. In *C. teleta*, we propose that *Ct-ngn* may act upstream in the neurogenic GRN, conferring a neural identity to ectodermal cells and maintaining them in a proliferative state, which is similar to the suggested role of a *neurogenin* homolog the annelid *P. dumerilii*. Our data further suggest that *Ct-ash1* turns on in a subset of *Ct-ngn*^+^ cells, possibly acting as a molecular switch promoting cell commitment and cell-cycle exit. Finally, we uncovered differences in the spatial localization of NPCs in the brain versus VNC in *C. teleta*, which when combined with previous evidence that neural fate specification is different between the brain and VNC, hints that these two neural tissues may represent different developmental modules, possibly with differing evolutionary trajectories. Overall, our data highlight the importance of understanding both cellular and molecular aspects of neurogenesis for a more holistic comparison across taxa.

## Methods

### Animal care

A laboratory colony of *Capitella teleta* adults were maintained in bowls of artificial seawater (ASW) and mud at 19 °C as described elsewhere [67, 116]. For all experiments, larvae were raised to the indicated developmental stages at 19 °C in ASW with 60 μg/mL penicillin and 50 μg/mL streptomycin (PenStrep) (Sigma Aldrich). Developmental stages were identified following a *C. teleta* staging chart described previously [67, 116].

### Lineage tracing of the 2d sublineage

The lipophilic dye DiIC_18_(3) (1,1′-dioctadecyl-3,3,3′,3′-tetramethylindocarbocyanine perchlorate, Invitrogen) was micro-injected into micromeres of the 2d sub-lineage (2d^1^, 2d^2^, 2d^11^ and 2d^112^) using aluminosilicate needles (Item no. AF100–64-10, Sutter Instruments Co.; O.D.–1.0 mm, I.D.–0.64 mm) pulled using the following parameters: HEAT = 400, FILAMENT = 4, VELOCITY = 60, DEL = 200, PUL = 170. Around 20–30 animals were injected for each 2d daughter blastomere analyzed per brood. A few injected embryos (*n* = 3) were kept aside for live time-lapse imaging while the rest were raised to specific developmental stages in ASW with PenStrep at 19 °C. Water was changed every 12–16 h to maintain the health of the injected animals. At specific time intervals, DiI-injected animals (*n* = 3) were fixed in 4% paraformaldehyde (PFA) (Electron Microscopy Sciences) and 0.05 M Ethylenediaminetetraacetic acid (EDTA) in ASW for 1 h at room temperature (r.t.). The fixative was removed with successive washes of phosphate buffered saline (PBS) and stained with 0.125 μg/ml Hoechst 33322 (1:1000) (Sigma) and 1:100 of 200 units/ml of BODIPY-Phallacidin (Invitrogen) in PBS overnight at 4 °C.

### Cell proliferation assays

Cell proliferation assays were performed using a combination of pulses, pulse-chases, and sequential pulse-chases with the thymidine analogs 5-Ethynyl-2′-deoxyuridine (EdU; ThermoFisher Cat# C10337) and 5-Bromo-2′-deoxyuridine (BrdU; ThermoFisher Cat# B23151). EdU labeling was conducted by incubating different stages of *C. teleta* embryos and larvae in 3 μM EdU diluted in ASW for 30 or 45 min at r.t. For EdU pulses, animals were fixed immediately after incubation in EdU. For pulse-chases, EdU was washed off chased briefly with thymidine for 3 h before allowing the animals to develop in ASW for different lengths of time before fixation in 4% PFA for 30 mins at r.t. For BrdU pulse-chase-wait-EdU sequential labeling, animals at stage 4 telotroch were incubated in BrdU for 2 h, grown in ASW until 24 or 48 h with a 3 h thymidine chase following BrdU incubation, incubated in EdU for 2 h, and then fixed in 4% PFA for 30 mins. All EdU incorporation was visualized using the Click-iT EdU Alexa Fluor 488 Imaging kit (ThermoFisher Cat# C10337). BrdU staining was developed using the mouse anti-BrdU antibody (3D4) (BD Biosciences) and subsequent immunostaining procedures (See Additional File 8: Supplementary Methods for details).

### Pretreatment before fixation

Prior to all fixation steps, *C. teleta* larval stages were subject to two types of pretreatment. 1) For stage 3 larval stages (no prototroch visible), animals were pretreated using a 1:1 mixture of freshly prepared 1 M sucrose and 0.25 M sodium citrate for 3 min followed by 2–3 ASW rinses and then fixation. 2) After prototroch formation, larvae at stages 4–6 were pretreated in a 1:1 mixture of 0.37 M MgCl_2_ and ASW for 5–10 min followed by fixation.

### Microscopy and image analysis

DiI-injected embryos were imaged live using an AxioCam MRm rev.3 camera and Zen Blue software (Zeiss) for 48–72 h at r.t. using time-lapse settings. A single embryo was mounted with a coverslip on a glass slide in a drop of ASW plus PenStrep surrounded by air and a ring of vacuum grease. Live Imaging for every blastomere assessed was conducted for 3 embryos across different broods. DIC and fluorescent z-stacks of 1 μm depth over 40 μm were automatically captured at every 1 to 1.5 h intervals for 48–72 h. All z-stacks were arranged in order and movies were created using Fiji (ImageJ2, NIH).

All fixed, DiI-injected animals, EdU labeling experiments, and FISH+EdU experiments were imaged using Zeiss IP-Apotome M2 with an AxioCam Mrm rev.3 camera. The BrdU pulse-chase-wait-EdU and double FISH experiments were imaged using a TCS SP5-X (Leica) confocal laser scanning microscope (CLSM). Z-projections were obtained, and stacks created from the CLSM using Fiji (ImageJ2, NIH). Transverse sections of the trunk were obtained using Fiji (ImageJ2, NIH) as well by projecting the imaged animal orthogonally along the Y-Z axis. Contrast and brightness of immunohistochemistry images were edited using Photoshop CC (Adobe) and figure panels were created using Illustrator CC (Adobe Systems Inc.).

### Cell counting

The number of cells in the developing head and trunk of *C. teleta* were counted using the Cell Counter plugin in Fiji (ImageJ2, NIH). For counting total number of EdU^+^ nuclei and EdU^+^/gene^+^ nuclei in the head, the “Grid” function in Fiji under Analyze - > Tools - > Grid was used. The animals were centered along the grid area and the total number of squares entirely spanning each of the brain lobes on the left and right sides were counted. This method took into account the presence of a considerable number of EdU^+^ nuclei and EdU^+^/gene^+^ nuclei along the lateral edges of the developing brain during stages 5 and 6. Counting was begun from the basal-most slices and continued apically to reduce re-counting of cells in the head. During this process the depth of the cells from the apical surface was not taken into account; only the total number of EdU^+^ cells and EdU^+^/gene^+^ cells were counted across the entire brain. For counting EdU^+^ cells after chase in the brain, we wanted to compare dynamics between apical versus basal populations as cells internalize from the apical surface. Due to the curvature of the head, surface cells located along the lateral edges tend to be detected as basally-localized (Additional File 2: Fig. S2c, dotted double-head arrows) using the method described above. A 30 μm × 30 μm area was defined and placed on the left and right sides of the brain near the midline to reduce such edge-effects originating from to the curved nature of the animal’s head. The region of interest [114] covered a major portion of the brain large enough to detect differences across stages. Within each ROI, the head was divided into 10 μm stacks from apical to basal and cells were counted within the defined area across four such stacks at different depths of the brain (0–10 μm, 10–20 μm, 20–30 μm, and 30–40 μm from the apical surface) (Additional File 2: Fig. S2c). Counting began from the basal-most stacks and continued apically in order to minimize recounting same cells and eliminating false positives. Similarly, the ROI was placed on the other brain lobe at a similar distance from the midline and the counting procedure was repeated. However, due to the curvature of the head, cells in ROIs 2 and 3 are not located at similar distances from the nearest apical surface, hence misrepresenting the data. For example, subsurface cells along the lateral edges of these ROIs can be misleading as they are actually apical (Additional File 2: Fig. S2c, dotted double-head arrows). However, for ROI 4, cells along the lateral edges are also well distant from the apical edge and hence represent basally-localized subsurface cells like the other cells in the same ROI. Therefore, to compare the number and proportion of EdU^+^ cells between apical and basal regions of the head, data from ROIs 2 and 3 were omitted from the plots but were used to calculate doubling rates of EdU^+^ cells in the head. We only plotted the comparative time-course dynamics between ROI 1 and ROI 4 that represent only entirely apical versus basal slices. Detailed cell counts across all ROIs can be found in Additional File 3: Table S7. Overall, although the numbers (counts and proportions) might not be reflective of the exact state at each stage, it still provides sufficient information to deduce trends of cell movement in the head. Using a smaller ROI did not provide information that was representative of the entire brain (Additional File 2: Fig. S2c).

As the depth of the trunk is smaller (i.e. ~ 20 μm), cells were counted within multiple 30 μm × 30 μm ROIs from anterior to posterior, each across the entire apical-to-basal depth within each ROI. All EdU, EdU pulse-chase and FISH+EdU counts were conducted using the following method elucidated below. In the trunk, as other tissues like the mesoderm are also developing underneath the ectoderm at the stages investigated, ROIs were placed on the presumptive neuroectoderm determined from the DiI labeling time-course experiment. As trunk segments are not visible until stage 5, at stage 4, the distance of the lateral DiI labeled neuroectodermal patch was averaged from the ventral midline across multiple animals. Using that measurement, ROI 1 was placed just below the mouth where future segments 2–4 will form and ROI 2 was placed immediately posterior to ROI 1. ROIs were placed closer to the medial edges of the neuroectoderm to avoid counting mesodermal cells (Additional File 7: Fig. S4h). A larger ROI extended more laterally and encroached into the mesodermal bands, hence a 30 μm × 30 μm ROI was specified. Stage 5 onwards, counts were conducted separately for anterior segments (segments 2–4) and posterior segments (segments 5–7) across both left and right sides of the trunk. The ROI was placed in a way such that one side touched the anterior boundary of segment 2 and the opposite side abutted into segment 4 for counting anterior segments. Similarly, for the posterior segments the ROI was aligned from the anterior boundary of segment 5 to segment 7 and subsequently segments 8–11 at later stages (Additional File 7: Fig. S4h). The posterior growth zone was avoided as there were way more dividing cells as compared to the other tissues.

### Statistical analysis

Different statistical approaches were used to test for changes in the numbers and proportions of EdU^+^ and EdU^+^/gene^+^ cells in the anterior and trunk neuroectoderm through time and in different ROIs.

The number and proportion of EdU^+^ and EdU^+^/gene^+^ cells in the anterior neuroectoderm obtained from static 30-min EdU labeling experiments and FISH+EdU experiments, respectively, were compared across stages 3–6 using a one-way ANOVA. We used number of EdU^+^ and EdU^+^/gene^+^ cells, total number of Hoescht^+^ cells and the proportion of EdU^+^ and EdU^+^/gene^+^ cells in the anterior neuroectoderm as our response variables that we compared across stages. We then used Tukey HSD posthoc tests to identify which developmental stages differed in these variables.

Experiments involving static 30-min EdU labeling in the trunk, FISH+EdU patterns in the trunk, and EdU pulse chases for both anterior and trunk neuroectoderm were statistically analyzed using mixed effects modeling. For the trunk static EdU counts, separate mixed effects models were run using number of EdU^+^ cells, total Hoescht^+^ cells and proportion of EdU^+^ cells relative to Hoescht^+^ cells as response variables. For these analyses, we include individuals, and trunk segment ROI nested in individual as random factors, with left and right sides represented in the residual random effects variation. We included developmental stage, ROI, and their interaction as fixed effects.

For the FISH+EdU experiments, we ran separate mixed effects models using total number of EdU^+^/gene^+^ cells and proportion of EdU^+^/gene^+^ cells relative to EdU^+^ cells for each of the three genes analyzed, *Ct-soxB1*, *Ct-ngn* and *Ct-ash1* as response variables. However, in these analysis ROI nested within individual did not explain any variation and was eliminated from the models. Hence individual was included as a random effect with measures for ROI and left and right sides captured in the residual random effects variation. Again, we included developmental stage, ROI, and their interaction as fixed effects.

We also used mixed effects modeling to analyze data from EdU pulse chase experiments for the head and trunk, but the design of these experiments differed from those described above. In these experiments, data were collected from six individuals per time point from each of three mothers (Additional File 8: Supplementary Methods). Cells were counted from each individual across several cell depths in the head and several segment groupings in the trunk. Data were also collected from the left and right sides at each depth or segment grouping. This nested pseudoreplication was accounted for by using mixed effects models for our analysis. The proportion of EdU^+^ cells relative to Hoechst^+^ cells, the number of EdU^+^ cells, and the number of Hoechst^+^ cells in the head and the trunk were response variables, for a total of six models. We included mother, individual nested within mother, and depth (for the head) or segment group (for the trunk) nested within individual within mother as random effects. The random effects residual variance accounted for data from the left and right sides. We then included timepoints (hours) post EdU pulse for each embryo, the depth or segment group, and their interaction as fixed effects.

We conducted all analyses in R v.3.5.1 [117]. We fitted the ANOVAs using the ‘aov’ function, and Tukey HSD post hoc tests using the ‘TukeyHSD’ function. We fitted the mixed effects models using the lme4 package [118]. We calculated effect sizes for the fixed and random effects by calculating marginal and conditional R^2^ using the MuMIn package [119]. Marginal R^2^ (R^2^_M_) is the proportion of variance in the response explained by only the fixed effects, and conditional R^2^ (R^2^_C_) is the proportion of variance explained by both fixed and random effects [119, 120]. We also calculated the percentage of the random effect variance explained by each random effect [121]. Finally, we conducted post hoc comparisons between a priori selected fixed effects levels using the emmeans package [122]. Specifically, we compared head depths or segment groups within each timepoint (hours) post EdU pulse, and all timepoints post EdU pulse within a head depth or segment group. We corrected *p*-values for multiple comparisons for each model while taking false discovery rate into account [123, 124]. Importantly, these comparisons took the random effects included in the model into account [125].

## Supplementary information

**Additional file 1: Figure S1.** Dynamics of cell proliferation in the anterior neuroectoderm. (A–D) 30-min EdU (green) pulses from stages 3–6. Arrows indicate EdU^+^ cells along the basal edges of the brain that are hypothesized to be part of the anterior mesoderm (E–G) 45-min EdU labeling coupled with anti-phospho-Histone H3 (PH3) immunostaining at stages 4–6. (H–J) Dot boxplots showing the dynamics of cell proliferation in the head across stages 3–6. Capital letters above the boxplots indicate statistical groups comparing cell counts or proportions at different stages. Boxplots with the same letter are not significantly different. Those with different letters are significantly different, with *p* < 0.05 after correction for multiple comparisons. Upper and lower bounds of the box plot indicate the 3rd and 1st quartiles while the middle line inside the boxplot indicates the median. The ends of the whiskers represent the 5th and 95th percentiles, black dots represent outliers (±3 S.D.). In A–G, the stages investigated are indicated at the lower left corner of each figure panel. ST3mo: stage 3 mouth, ST4pt: stage 4 prototroch, ST4tt: stage 4 telotroch, ST5: stage 5, ST6: stage 6. Scale bar: 50 μm.

**Additional file 2: Figure S2.** (A) EdU pulse-chase experiments involving 30-min EdU pulses at stage 4 once a telotroch is present followed by 3-h thymidine chase and incubation in seawater for different lengths of time as indicated. (B) Images showing apical to basal migration of initially labeled NPCs and their daughters in the head with time. Length of time post-EdU pulse indicated on the upper-left corner of each panel (C) Method for counting EdU^+^ cells using ROIs across different depths of the head. Differentially colored boxes indicate the ROIs at different depths where counts were conducted. The solid double-head arrow indicates distance between each ROI. The dotted double-head arrow indicates the distance of the lateral edges of the ROIs from the nearest apical surface showing that cells lying in those regions can misleadingly represent basally-localized cells when they are actually apical. (D–F) Graphs indicate the dynamics and behavior of initially labeled NPCs and their daughters across different lengths of time. The x-axis represents the times post EdU pulse while the panels indicate depths for ROI 1 and 4. Boxplots within each depth (ROI 1 versus 4) indicate the lengths of the seawater chase in ascending order beginning from left to right – 0 h (red) and 48 h (blue). Capital letters above the boxplots compare chase within a particular ROI and hence are comparisons across each set of eight adjacent box plots. The lowercase letters below the boxplots indicate comparison of apical versus basal depths (ROI 1 versus ROI 4) at a particular time point e.g. comparison between the first boxplot of the two different depths and so on (for example, 0–10 μm depth at t = 0 versus 30–40 μm depth at t = 0). In all cases, “a” or “A” corresponds to treatments that have the lowest values and then the letters advance in alphabetical order as the y-axis variable increases. Boxplots with the same letter are not significantly different. Those with different letters are significantly different, with p < 0.05 after correction for multiple comparisons. ST4tt: stage 4 telotroch, ST5e: stage 5 early, ST5mid: stage 5 middle, ST5l: Stage 5 late, ST6e: stage 6 early, ST6mid: stage 6 mid. Scale bar: 50 μm.

**Additional file 3: Table S7.** Detailed EdU pulse-chase counts in the anterior neuroectoderm across 0–48 h.

**Additional file 4: Figure S3.** Fates of subclones derived from the 2d sub-lineage. (A–L’) Apotome micrographs of *C. teleta* at different stages of neurogenesis (0–72 h post injection (hpi)) labeled with DiI (red) and Hoescht33322 (cyan) derived from 2d^11^ (A–D’), 2d^1^ (E–H′) and 2d^2^ (I–L’). Closed and open arrowheads in H show cell intercalation between DiI^+^ cells from the right side and Hoescht labeled nuclei from the left side. Closed arrowheads indicate a DiI^+^ cell whereas an open arrowhead indicates an intercalated non-DiI^+^. Asterisk in panels A, A’, E, E’, I and I′ indicates the blastopore while in all other panels asterisk denotes the mouth opening. A, A’, E, E’, I and I′ panels indicate vegetal views of blastopore stage 3 while all other panels are ventral views. In each panel, anterior is to the left and posterior the right. The number of animals examined and showing the staining pattern is indicted on the top right-hand corner of each panel. Bottom rows indicate DiI labeled patches in black and white. Prototroch (pt) and telotroch (tt) are indicated by dashes. The length of time each animal is grown is indicated at the lower left corner. Vnc: ventral nerve cord, nt: neurotroch, nec: neuroectoderm, pt.: prototroch, tt: telotroch, pg: pygidium, veg: vegetal. Scale bar: 50 μm.

**Additional file 5: Movie S1.** Time-lapse video showing progression of 2d^112^ -derived trunk neuroectodermal boundaries. Video shows a stage 4 larvae (48 hpi) injected with DiI (red) at 64–128 cell stage and imaged every 1.5 h at a speed of 3 frames per second (fps). DiI stained animals are shown here in black and white with the DiI stained tissue shown in white and the background being the non-stained tissue.

**Additional file 6: Movie S2.** Time-lapse video showing progression of 2d^11^ -derived cell populations in the trunk. Video shows a stage 4 larvae (48 hpi) injected with DiI (red) at 32–64 cell stage and imaged every 1 h at a speed of 3 frames per second (fps). DiI labeled animals are shown here in black and white with DiI stained tissue shown in white and the background being the non-stained tissue.

**Additional file 7: Figure S4.** Assessment of cell proliferation and contribution of EdU^+^ NPCs at stage 4 to the VNC. (A) Schematic showing EdU pulse chase experiment with EdU pulse at stage 4 telotroch followed by 3 h of 10 μm thymidine chase and subsequent incubation in sea-water for respective time lengths. (B–G.2) Panels show the cell proliferation profiles and the behavior of their progeny from 0 h till 72 h. Ventral views (B, C, D, E, F, G) and orthogonal views (B.1, B.2, C.1, C.2, D.1, D.2, E.1, E.2, F.1, F.2, G.1, G.2) of larval trunk neuroectoderm at six different time intervals (0 h, 6 h, 9 h, 20 h, 36 h and 72 h) shown labeled with EdU (green) and Hoescht 33,322 (magenta). B.1, C.1, D.1. E.1, F.1, G.1 indicate orthogonal views along the dashed line labeled “1” and B.2, C.2, D.2. E.2, F.2, G.2 represent orthogonal views along the dashed line labeled “2” in B, C, D, E, F, G, respectively. Arrows in D.2, E.1, E.2, F.1, F.2 indicate stippled labeled EdU^+^ cells localized on the surface trunk ectoderm. In each panel showing ventral views (B, C, D, E, F, G), anterior is to the left and posterior the right. Prototroch (pt) and telotroch (tt) are indicated by dashes. Asterisk denotes the position of the mouth in all ventral views. The length of thymidine chase and sea-water incubation is indicated at the upper right-hand corner. In the orthogonal views, the yellow dot denotes the position of the ventral midline. Apical is upwards while basal is down in all orthogonal views (B.1, B.2, C.1, C.2, D.1, D.2, E.1, E.2, F.1, F.2, G.1, G.2). (H) Counting method for EdU^+^ cells and Hoescht^+^ cells in the trunk. For stage 4, the distance of the presumptive neuroectoderm was measured to be ~ 22 μm from the ventral midline (dotted line segment). Square boxes represent 30 μm × 30 μm ROIs where cells were counted using Fiji Cell-Counter plugin (ImageJ, NIH). In each animal ROIs 1 and 2 were counted on the left and right sides of the animal for stages 4 and 5. For stage 6, ROIs 1, 2 and 3 were counted on either side of the midline. ST4tt: stage 4 telotroch, ST5e: stage 5 early, ST5mid: stage 5 middle, ST5l: stage 5 late, ST6e: stage 6 early, ST6mid: stage 6 middle. mo: mouth, ms: mesoderm, nec: neuroectoderm. Scale bar: 50 μm.

**Additional file 8. Supplementary Material** Supplementary Information with Supplementary Methods and Tables S1–S6.

**Additional file 9: Table S8.** Detailed EdU pulse-chase counts in the trunk neuroectoderm across 0–48 h.

**Additional file 10: Figure S5.** Gene expression in trunk EdU^+^ cells at later developmental stages. (A–E”) 30-min EdU pulse (cyan) at different stages of neurogenesis was combined with FISH (red). Ventral views of the trunk neuroectoderm (A, B, C, D, E) and transverse sections through the anterior (A’, B′, C′, D’, E’), and posterior (A”, B″, C″, D”, E”) trunk neuroectoderm are shown. Overlap of EdU^+^ cells with *Ct-soxB1* (A–A”), *Ct-ngn* (B–B″), *Ct-ash1* (C–C″), *Ct-neuroD* (D–D”), and *Ct-elav1* (E–E”) at later stages are shown. In panels A–E”, closed arrowheads indicate EdU^+^/gene^+^ cells while open arrowheads show EdU^−^/gene^+^ cells. In ventral view panels A–E, orientation of the animal is indicated in the bottom right corner, and developmental stage is indicated in the top right corner. (F–H) The number of EdU^+^ cells expressing a *Ct-soxB1* (F), *Ct-ngn* (G), and *Ct-ash1* (H) counted within ROI 1, 2 and 3 in segments 2–4, 5–7, and 8–10 in the VNC were scored at stages 4–6 as shown in Fig. S4H. In F–H, capital letters on top of the boxplots (e.g. A, B etc.) indicate statistical significance computed using mixed effects model analysis for comparison of individual ROIs across stages, e.g., comparison of EdU^+^/gene^+^ numbers in ROI 1 at stage 4 to that at stage 5 and 6 and so on for ROI 2. Black dots represent outliers (±3 S.D.). In orthogonal views, apical is up, basal is down and yellow dot shows the position of the ventral midline. White dotted line marks the apical boundaries of the neuroectoderm. In ventral views, anterior is to the left and posterior to the right. An asterisk denotes the mouth. Scale bar is 50 μm. ant, anterior; vent, ventral. ST4mid: Stage 4 middle, ST4tt: Stage 4 telotroch, ST5: Stage 5, ST6: Stage 6.

**Additional file 11: Figure S6.** Spatial localization of neurogenic homologs in the trunk neuroectoderm. (A–C) a subset of *Ct-soxB1*^+^ cells (cyan) which express *Ct-ngn* (red) in the presumptive neuroectoderm at stage 4. (D–F) *Ct-ngn* (cyan) is expressed over a broad domain in the trunk ectoderm while *Ct-ash1* (red) is expressed in a punctate manner throughout the trunk ectoderm, only sometimes in *Ct-ngn*^*+*^ cells. (G–I) *Ct-elav1* (cyan) and *Ct-ash1* (red) are expressed in non-overlapping domains in the trunk. C′, C″, F′, F″, I′, I″ are orthogonal views through anterior (C′, F′, I′) and posterior (C″, F″, I″) segments of the trunk neuroectoderm. The asterisk indicates the position of the mouth. In all orthogonal views apical is up and the yellow dot denotes the ventral midline. Dashed line indicates the apical edge of the transverse sections. In all figure panels, closed arrowheads indicate co-expression of two neurogenic homologs in surface cells while open arrowheads indicate that in sub-surface cells in respective panels. Arrows indicate non-overlapping expression of neurogenic homologs. Orientation of images are indicated on the lower right corner. The different developmental stages investigated are indicated at the lower left corner of each figure panel. Vent, ventral. ST4: Stage 4, ST6: Stage 6, ST7: Stage 7. Scale bar: 25 μm.

## Data Availability

The datasets used and/or analyzed during the current study are available from the corresponding author on reasonable request.

## Abbreviations

ANOVA: Analysis of variance
Ash: Achaete-scute homolog
ASW: Artificial sea-water
bHLH: Basic helix-loop-helix
BrdU: 5-Bromo-2′-deoxyuridine
CLSM: Confocal laser scanning microscopy
CNS: Central nervous system
DIC: Differential interference contrast
DiI: 1,1’-dioctadecyl-3,3,3′,3′-tetramethylindocarbocyanine perchlorate
dFISH: Double fluorescent in-situ hybridization
EdU: 5-Ethynyl-2′-deoxyuridine
FISH: Fluorescent in-situ hybridization
GRN: Gene regulatory network
Ngn: Neurogenin
NPC: Neural precursor cell
NS: Nervous system
PH3: Phosphorylated histone 3
ROI: Region of interest
VNC: Ventral nerve cord
