# Supplementary figures and images for: Investigating cellular and molecular mechanisms of neurogenesis in Capitella teleta sheds light on the ancestor of Annelida
Source: BMC Evol Biol. 2020 Jul 14;20:84. doi: 10.1186/s12862-020-01636-1 (PMC7362552; doi:10.1186/s12862-020-01636-1)

Static 30min **EdU** pulse + **Hoescht**

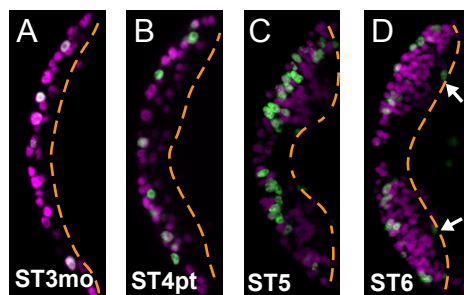

Static 45min **EdU** pulse + **phospho-histone** immunostaining

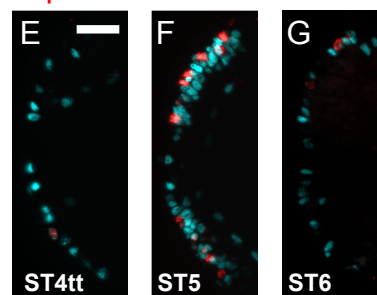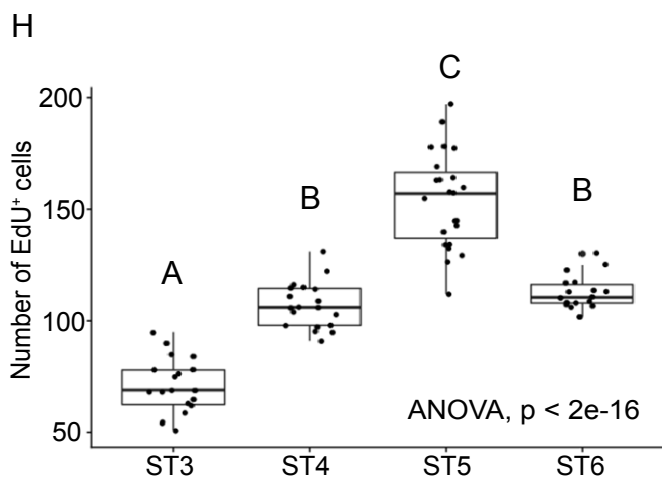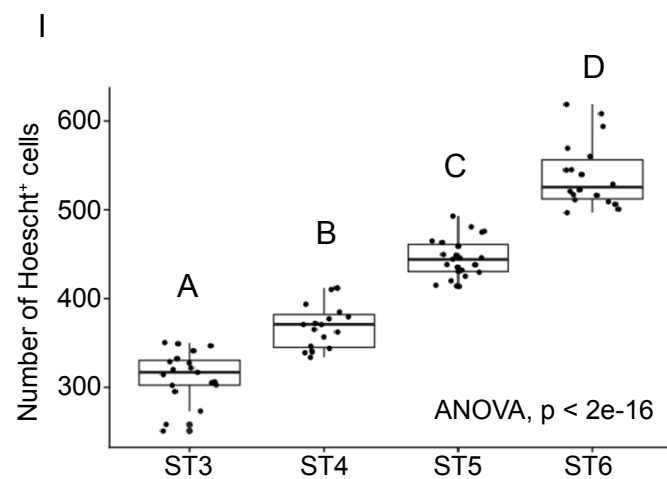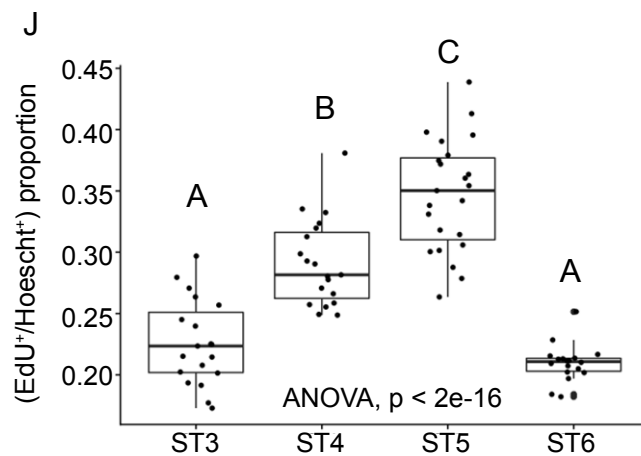

Supplement: Supplementary file 1 — Additional file 1: Figure S1. Dynamics of cell proliferation in the anterior neuroectoderm. (A–D) 30-min EdU (green) pulses from stages 3–6. Arrows indicate EdU+ cells along the basal edges of the brain that are hypothesized to be part of the anterior mesoderm (E–G) 45-min EdU labeling coupled with anti-phospho-Histone H3 (PH3) immunostaining at stages 4–6. (H–J) Dot boxplots showing the dynamics of cell proliferation in the head across stages 3–6. Capital letters above the boxplots indicate statistical groups comparing cell counts or proportions at different stages. Boxplots with the same letter are not significantly different. Those with different letters are significantly different, with p < 0.05 after correction for multiple comparisons. Upper and lower bounds of the box plot indicate the 3rd and 1st quartiles while the middle line inside the boxplot indicates the median. The ends of the whiskers represent the 5th and 95th percentiles, black dots represent outliers (±3 S.D.). In A–G, the stages investigated are indicated at the lower left corner of each figure panel. ST3mo: stage 3 mouth, ST4pt: stage 4 prototroch, ST4tt: stage 4 telotroch, ST5: stage 5, ST6: stage 6. Scale bar: 50 μm. [file 12862_2020_1636_MOESM1_ESM.pdf]

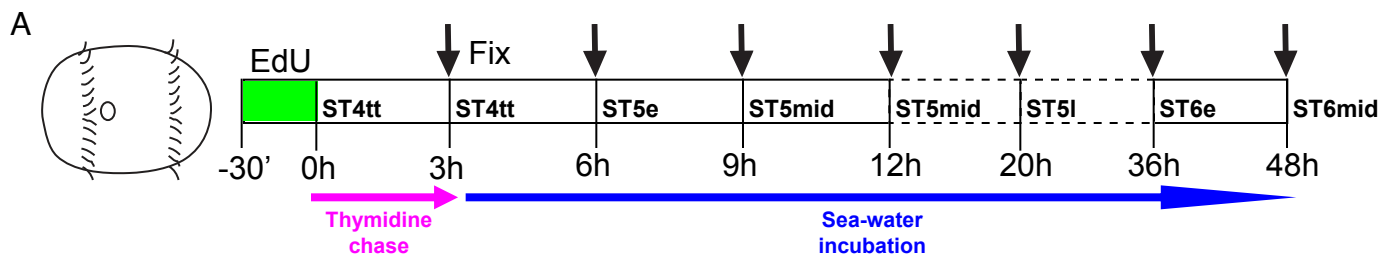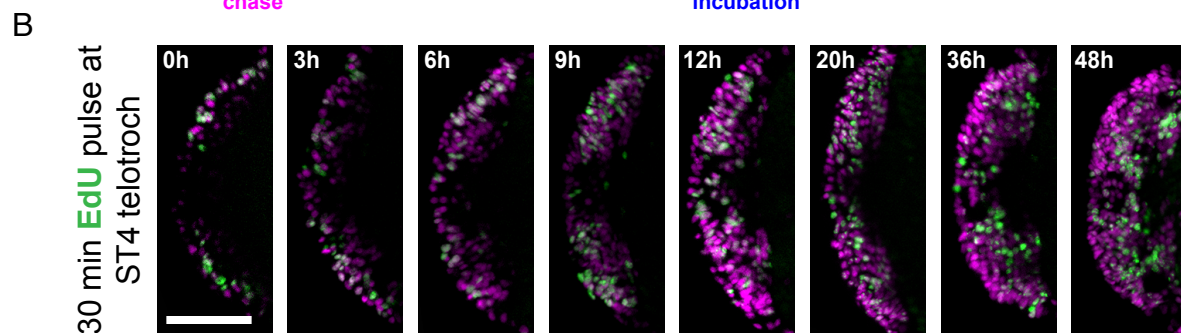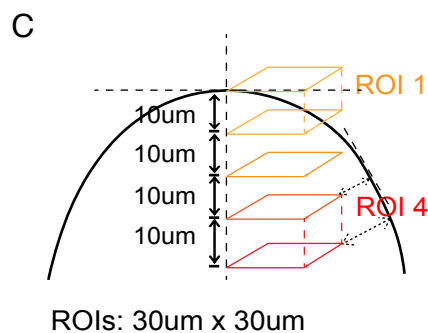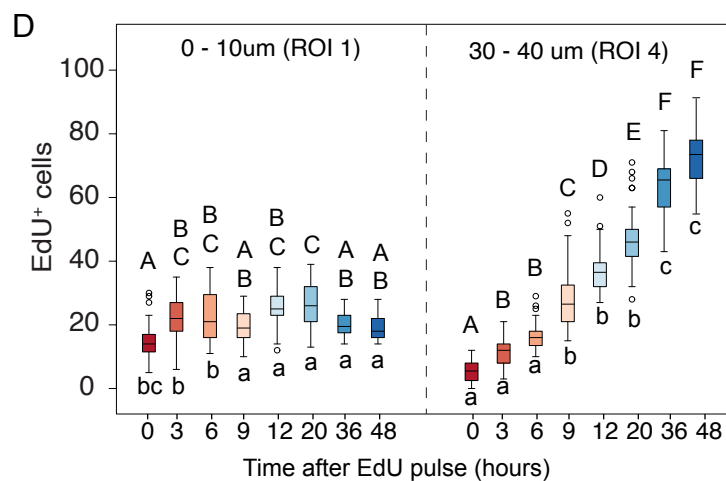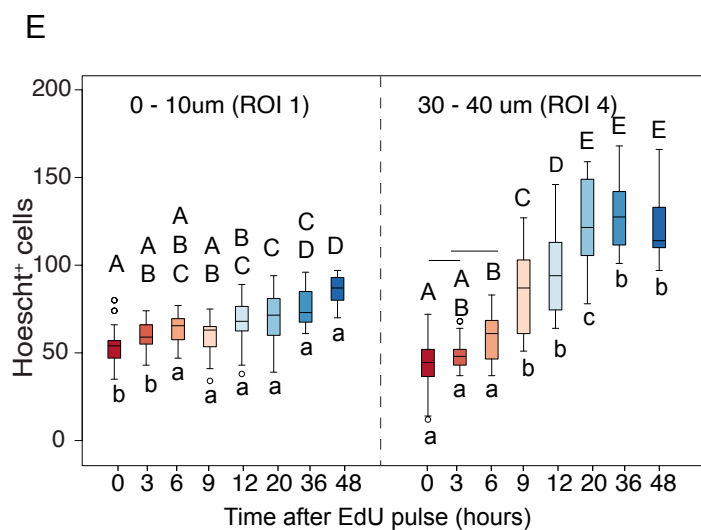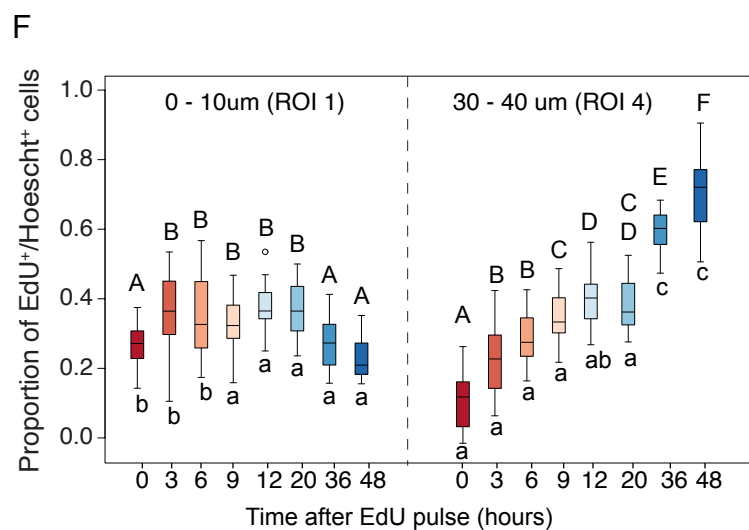

Supplement: Supplementary file 2 — Additional file 2: Figure S2. (A) EdU pulse-chase experiments involving 30-min EdU pulses at stage 4 once a telotroch is present followed by 3-h thymidine chase and incubation in seawater for different lengths of time as indicated. (B) Images showing apical to basal migration of initially labeled NPCs and their daughters in the head with time. Length of time post-EdU pulse indicated on the upper-left corner of each panel (C) Method for counting EdU+ cells using ROIs across different depths of the head. Differentially colored boxes indicate the ROIs at different depths where counts were conducted. The solid double-head arrow indicates distance between each ROI. The dotted double-head arrow indicates the distance of the lateral edges of the ROIs from the nearest apical surface showing that cells lying in those regions can misleadingly represent basally-localized cells when they are actually apical. (D–F) Graphs indicate the dynamics and behavior of initially labeled NPCs and their daughters across different lengths of time. The x-axis represents the times post EdU pulse while the panels indicate depths for ROI 1 and 4. Boxplots within each depth (ROI 1 versus 4) indicate the lengths of the seawater chase in ascending order beginning from left to right – 0 h (red) and 48 h (blue). Capital letters above the boxplots compare chase within a particular ROI and hence are comparisons across each set of eight adjacent box plots. The lowercase letters below the boxplots indicate comparison of apical versus basal depths (ROI 1 versus ROI 4) at a particular time point e.g. comparison between the first boxplot of the two different depths and so on (for example, 0–10 μm depth at t = 0 versus 30–40 μm depth at t = 0). In all cases, “a” or “A” corresponds to treatments that have the lowest values and then the letters advance in alphabetical order as the y-axis variable increases. Boxplots with the same letter are not significantly different. Those with different letters are signific [file 12862_2020_1636_MOESM2_ESM.pdf]

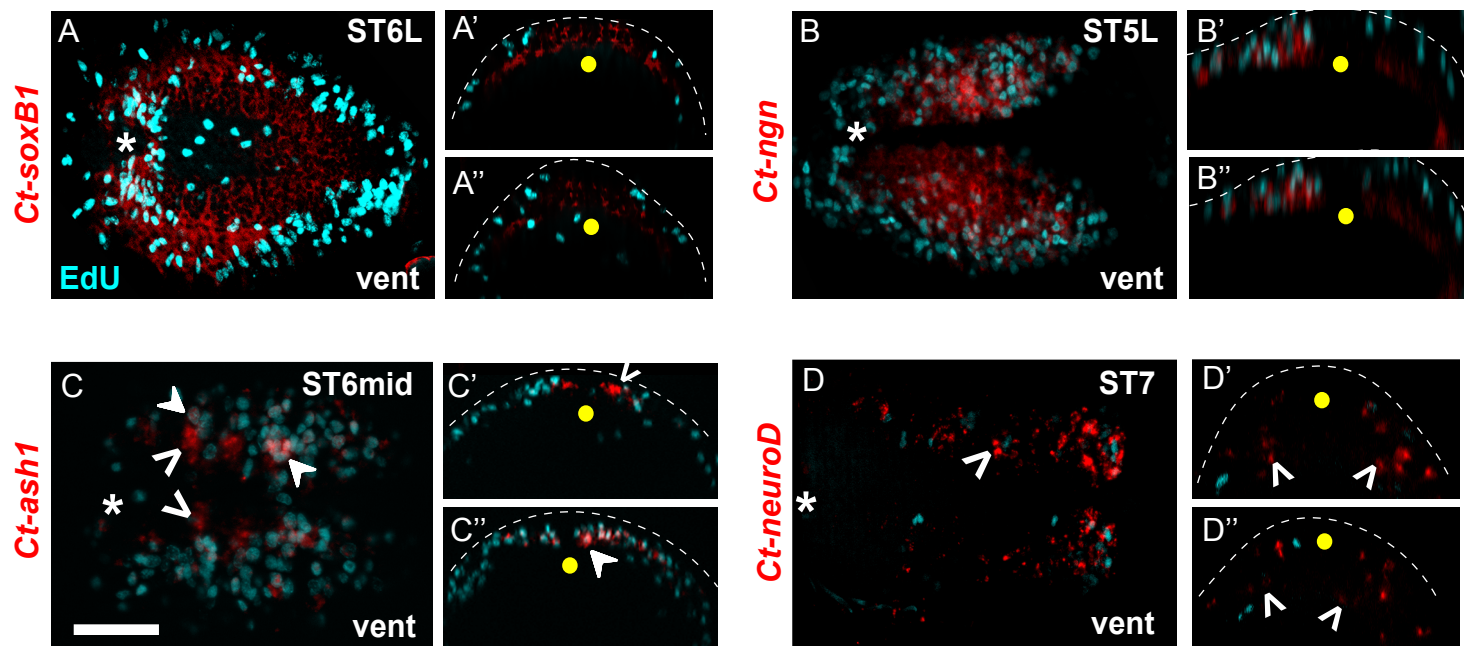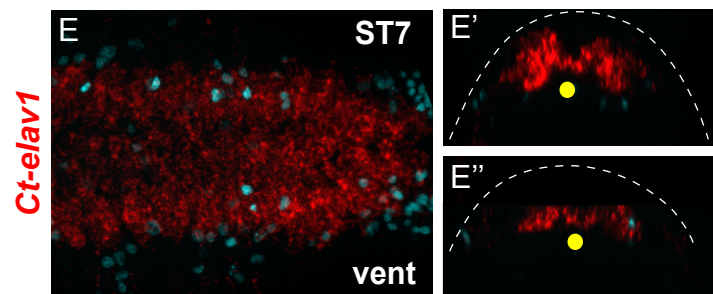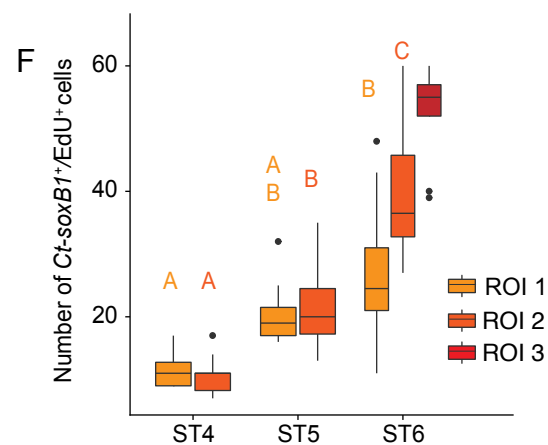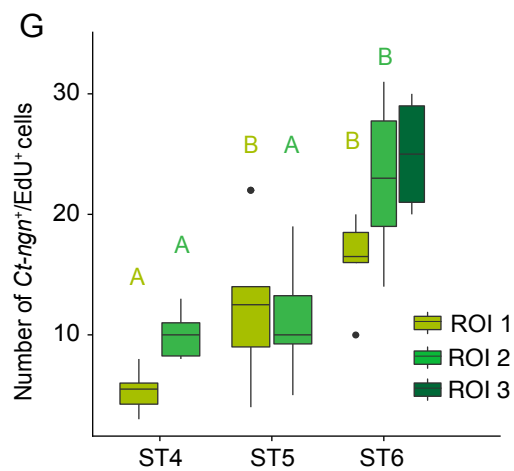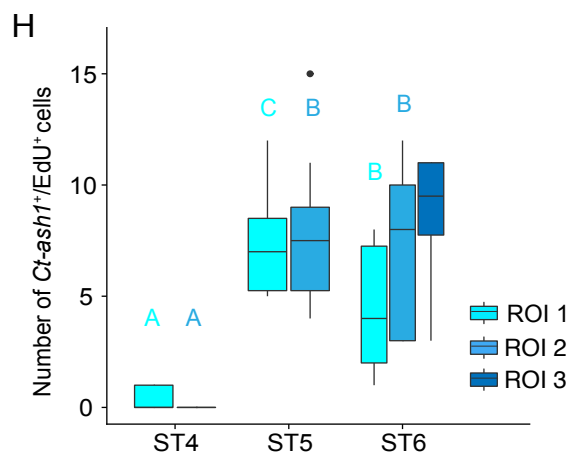

Supplement: Supplementary file 10 — Additional file 10: Figure S5. Gene expression in trunk EdU+ cells at later developmental stages. (A–E”) 30-min EdU pulse (cyan) at different stages of neurogenesis was combined with FISH (red). Ventral views of the trunk neuroectoderm (A, B, C, D, E) and transverse sections through the anterior (A’, B′, C′, D’, E’), and posterior (A”, B″, C″, D”, E”) trunk neuroectoderm are shown. Overlap of EdU+ cells with Ct-soxB1 (A–A”), Ct-ngn (B–B″), Ct-ash1 (C–C″), Ct-neuroD (D–D”), and Ct-elav1 (E–E”) at later stages are shown. In panels A–E”, closed arrowheads indicate EdU+/gene+ cells while open arrowheads show EdU−/gene+ cells. In ventral view panels A–E, orientation of the animal is indicated in the bottom right corner, and developmental stage is indicated in the top right corner. (F–H) The number of EdU+ cells expressing a Ct-soxB1 (F), Ct-ngn (G), and Ct-ash1 (H) counted within ROI 1, 2 and 3 in segments 2–4, 5–7, and 8–10 in the VNC were scored at stages 4–6 as shown in Fig. S4H. In F–H, capital letters on top of the boxplots (e.g. A, B etc.) indicate statistical significance computed using mixed effects model analysis for comparison of individual ROIs across stages, e.g., comparison of EdU+/gene+ numbers in ROI 1 at stage 4 to that at stage 5 and 6 and so on for ROI 2. Black dots represent outliers (±3 S.D.). In orthogonal views, apical is up, basal is down and yellow dot shows the position of the ventral midline. White dotted line marks the apical boundaries of the neuroectoderm. In ventral views, anterior is to the left and posterior to the right. An asterisk denotes the mouth. Scale bar is 50 μm. ant, anterior; vent, ventral. ST4mid: Stage 4 middle, ST4tt: Stage 4 telotroch, ST5: Stage 5, ST6: Stage 6. [file 12862_2020_1636_MOESM10_ESM.pdf]

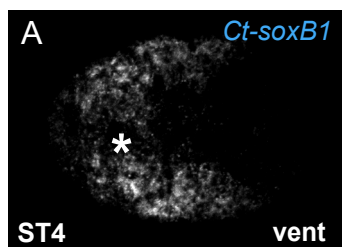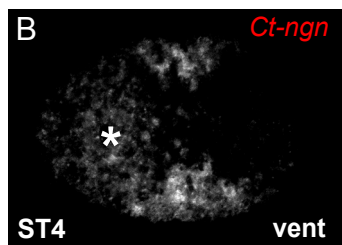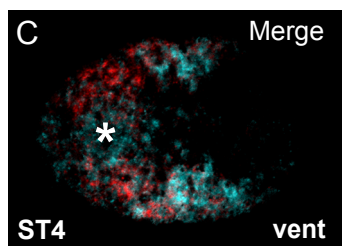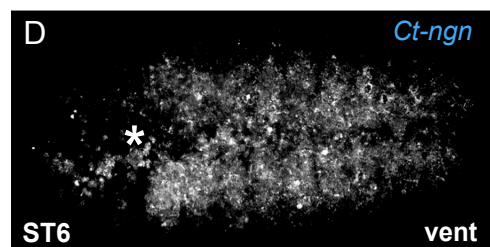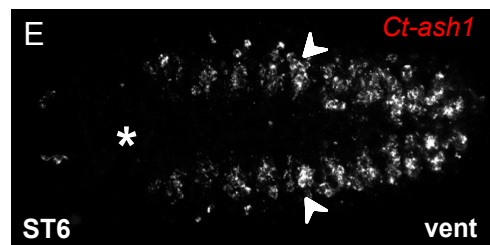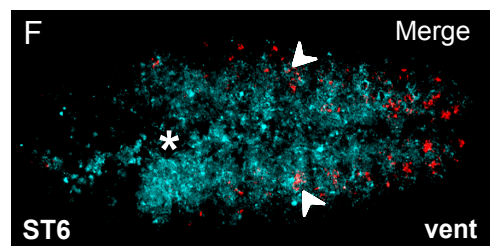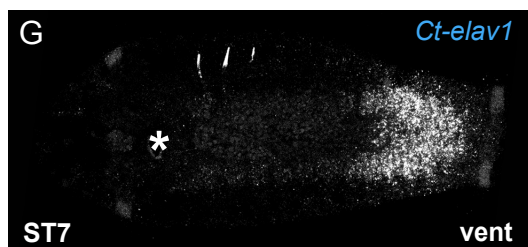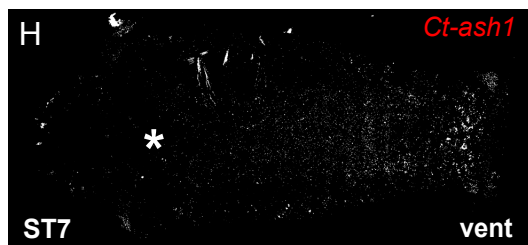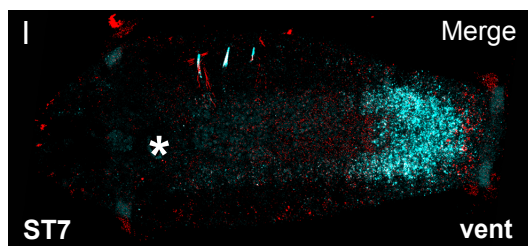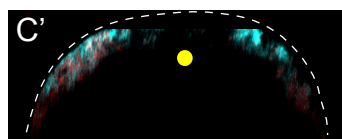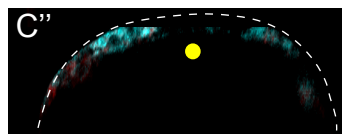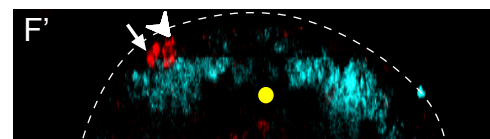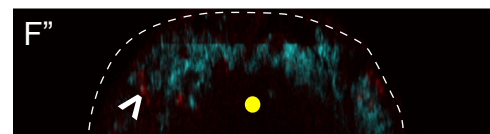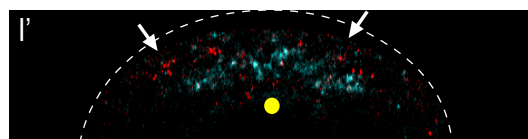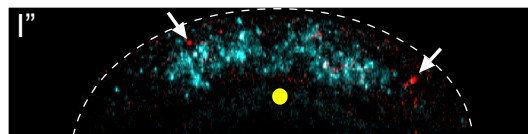

Supplement: Supplementary file 11 — Additional file 11: Figure S6. Spatial localization of neurogenic homologs in the trunk neuroectoderm. (A–C) a subset of Ct-soxB1+ cells (cyan) which express Ct-ngn (red) in the presumptive neuroectoderm at stage 4. (D–F) Ct-ngn (cyan) is expressed over a broad domain in the trunk ectoderm while Ct-ash1 (red) is expressed in a punctate manner throughout the trunk ectoderm, only sometimes in Ct-ngn+ cells. (G–I) Ct-elav1 (cyan) and Ct-ash1 (red) are expressed in non-overlapping domains in the trunk. C′, C″, F′, F″, I′, I″ are orthogonal views through anterior (C′, F′, I′) and posterior (C″, F″, I″) segments of the trunk neuroectoderm. The asterisk indicates the position of the mouth. In all orthogonal views apical is up and the yellow dot denotes the ventral midline. Dashed line indicates the apical edge of the transverse sections. In all figure panels, closed arrowheads indicate co-expression of two neurogenic homologs in surface cells while open arrowheads indicate that in sub-surface cells in respective panels. Arrows indicate non-overlapping expression of neurogenic homologs. Orientation of images are indicated on the lower right corner. The different developmental stages investigated are indicated at the lower left corner of each figure panel. Vent, ventral. ST4: Stage 4, ST6: Stage 6, ST7: Stage 7. Scale bar: 25 μm. [file 12862_2020_1636_MOESM11_ESM.pdf]
