# Supplementary material for: Investigating cellular and molecular mechanisms of neurogenesis in Capitella teleta sheds light on the ancestor of Annelida
Source: BMC Evol Biol. 2020 Jul 14;20:84. doi: 10.1186/s12862-020-01636-1 (PMC7362552; doi:10.1186/s12862-020-01636-1)
Supplement: Supplementary file 4 — Additional file 4: Figure S3. Fates of subclones derived from the 2d sub-lineage. (A–L’) Apotome micrographs of C. teleta at different stages of neurogenesis (0–72 h post injection (hpi)) labeled with DiI (red) and Hoescht33322 (cyan) derived from 2d11 (A–D’), 2d1 (E–H′) and 2d2 (I–L’). Closed and open arrowheads in H show cell intercalation between DiI+ cells from the right side and Hoescht labeled nuclei from the left side. Closed arrowheads indicate a DiI+ cell whereas an open arrowhead indicates an intercalated non-DiI+. Asterisk in panels A, A’, E, E’, I and I′ indicates the blastopore while in all other panels asterisk denotes the mouth opening. A, A’, E, E’, I and I′ panels indicate vegetal views of blastopore stage 3 while all other panels are ventral views. In each panel, anterior is to the left and posterior the right. The number of animals examined and showing the staining pattern is indicted on the top right-hand corner of each panel. Bottom rows indicate DiI labeled patches in black and white. Prototroch (pt) and telotroch (tt) are indicated by dashes. The length of time each animal is grown is indicated at the lower left corner. Vnc: ventral nerve cord, nt: neurotroch, nec: neuroectoderm, pt.: prototroch, tt: telotroch, pg: pygidium, veg: vegetal. Scale bar: 50 μm. [file 12862_2020_1636_MOESM4_ESM.pdf]

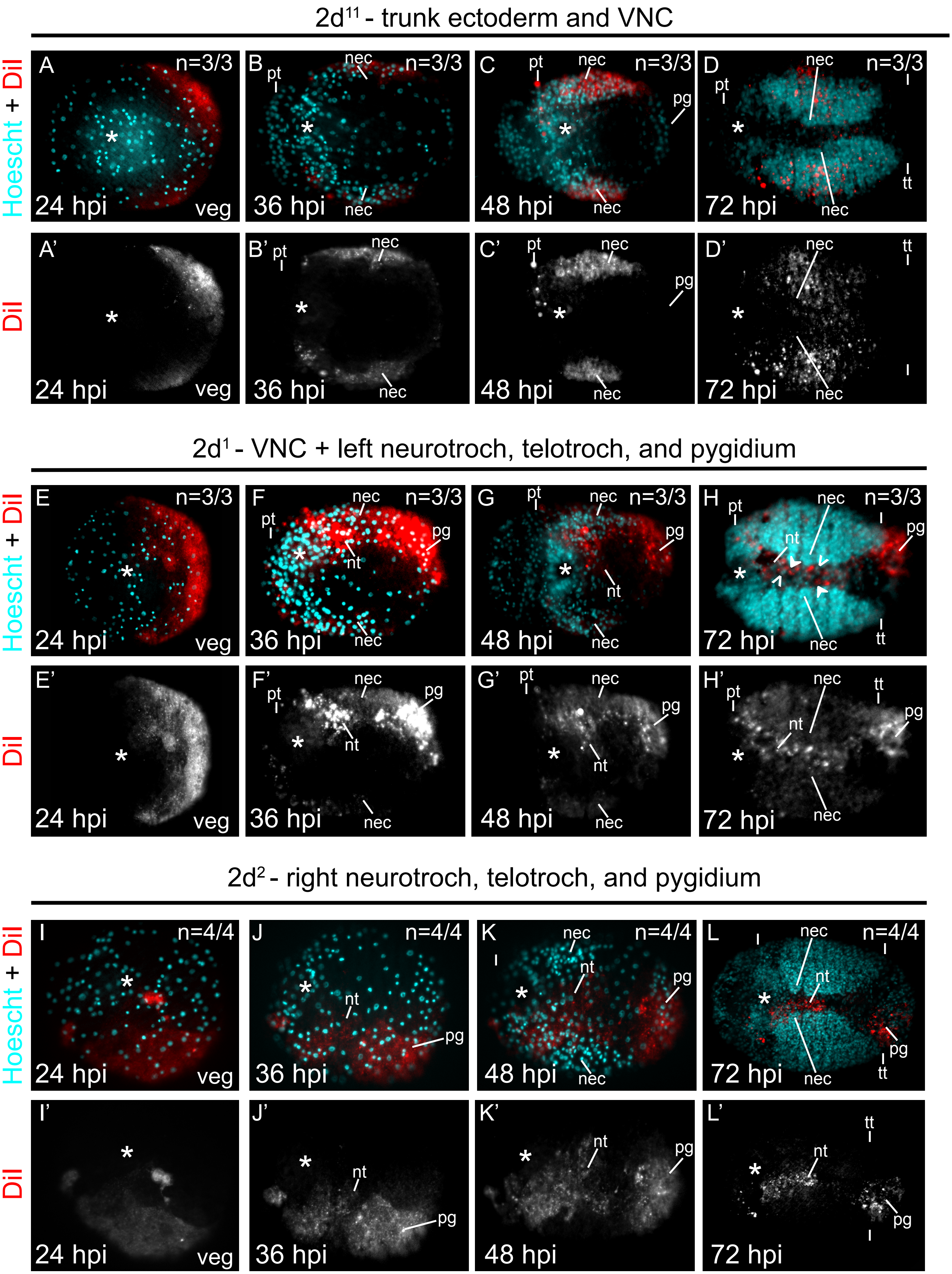

2d11 - trunk ectoderm and VNC
2d1 - VNC + left neurotroch, telotroch, and pygidium
2d2 - right neurotroch, telotroch, and pygidium
Hoescht + DiI
DiI
A B C D A' B' C' D'
E F G H E' F' G' H'
I J K L I' J' K' L'
n=3/3
n=4/4
24 hpi
36 hpi
48 hpi
72 hpi
veg
pt
nec
pg
tt
nt
