# Supplementary material for: Investigating cellular and molecular mechanisms of neurogenesis in Capitella teleta sheds light on the ancestor of Annelida
Source: BMC Evol Biol. 2020 Jul 14;20:84. doi: 10.1186/s12862-020-01636-1 (PMC7362552; doi:10.1186/s12862-020-01636-1)
Supplement: Supplementary file 7 — Additional file 7: Figure S4. Assessment of cell proliferation and contribution of EdU+ NPCs at stage 4 to the VNC. (A) Schematic showing EdU pulse chase experiment with EdU pulse at stage 4 telotroch followed by 3 h of 10 μm thymidine chase and subsequent incubation in sea-water for respective time lengths. (B–G.2) Panels show the cell proliferation profiles and the behavior of their progeny from 0 h till 72 h. Ventral views (B, C, D, E, F, G) and orthogonal views (B.1, B.2, C.1, C.2, D.1, D.2, E.1, E.2, F.1, F.2, G.1, G.2) of larval trunk neuroectoderm at six different time intervals (0 h, 6 h, 9 h, 20 h, 36 h and 72 h) shown labeled with EdU (green) and Hoescht 33,322 (magenta). B.1, C.1, D.1. E.1, F.1, G.1 indicate orthogonal views along the dashed line labeled “1” and B.2, C.2, D.2. E.2, F.2, G.2 represent orthogonal views along the dashed line labeled “2” in B, C, D, E, F, G, respectively. Arrows in D.2, E.1, E.2, F.1, F.2 indicate stippled labeled EdU+ cells localized on the surface trunk ectoderm. In each panel showing ventral views (B, C, D, E, F, G), anterior is to the left and posterior the right. Prototroch (pt) and telotroch (tt) are indicated by dashes. Asterisk denotes the position of the mouth in all ventral views. The length of thymidine chase and sea-water incubation is indicated at the upper right-hand corner. In the orthogonal views, the yellow dot denotes the position of the ventral midline. Apical is upwards while basal is down in all orthogonal views (B.1, B.2, C.1, C.2, D.1, D.2, E.1, E.2, F.1, F.2, G.1, G.2). (H) Counting method for EdU+ cells and Hoescht+ cells in the trunk. For stage 4, the distance of the presumptive neuroectoderm was measured to be ~ 22 μm from the ventral midline (dotted line segment). Square boxes represent 30 μm × 30 μm ROIs where cells were counted using Fiji Cell-Counter plugin (ImageJ, NIH). In each animal ROIs 1 and 2 were counted on the left and right sides of the animal for stages 4 and 5. For stage 6, ROIs 1, 2 [file 12862_2020_1636_MOESM7_ESM.pdf]

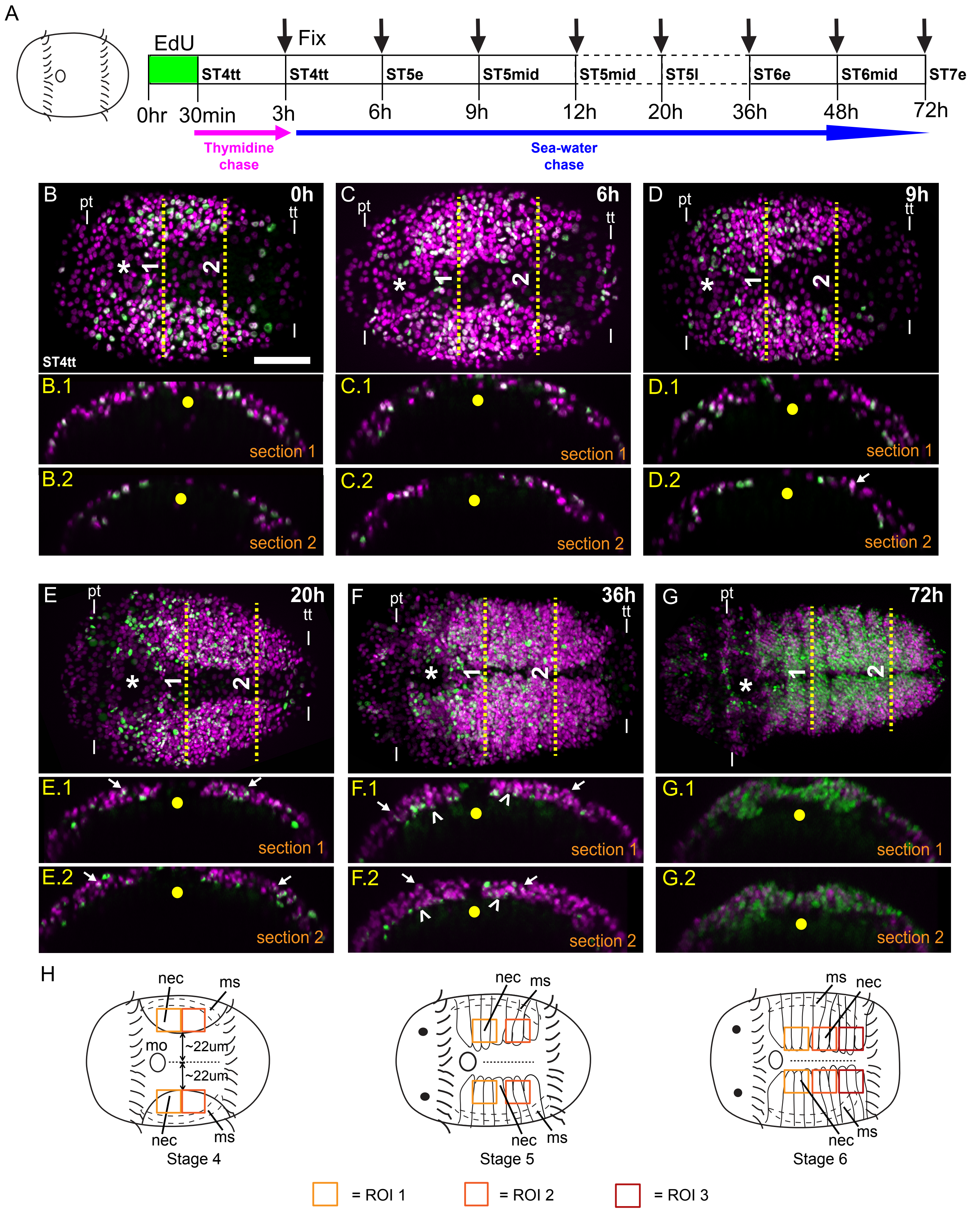

A
EdU
Fix
ST4tt
ST4tt
ST5e
ST5mid
ST5mid
ST5l
ST6e
ST6mid
ST7e
0hr
30min
3h
6h
9h
12h
20h
36h
48h
72h
Thymidine chase
Sea-water chase
B
0h
pt
tt
ST4tt
C
6h
D
9h
B.1
section 1
B.2
section 2
C.1
C.2
D.1
D.2
E
20h
F
36h
G
72h
E.1
E.2
F.1
F.2
G.1
G.2
H
nec
ms
mo
~22um
~22um
Stage 4
Stage 5
Stage 6
= ROI 1
= ROI 2
= ROI 3
