# Supplementary material for: Investigating cellular and molecular mechanisms of neurogenesis in Capitella teleta sheds light on the ancestor of Annelida
Source: BMC Evol Biol. 2020 Jul 14;20:84. doi: 10.1186/s12862-020-01636-1 (PMC7362552; doi:10.1186/s12862-020-01636-1)
Supplement: Supplementary file 8 — Additional file 8. Supplementary Material Supplementary Information with Supplementary Methods and Tables S1–S6. [file 12862_2020_1636_MOESM8_ESM.docx]

**Supplementary Information**

**Supplementary Methods:**

**Cell Proliferation Assays**

1. **Simple EdU labeling and PH3+ immunostaining**

For simple EdU incorporation, animals at stages 3-6 randomly chosen from a pool of embryos collected from multiple mothers were exposed to 3 µM EdU (5-Ethynyl-2′-deoxyuridine) in 0.2 μm ASW for 30 mins at room temperature (r.t.). Following incubation, animals were washed with 0.2 μm ASW thrice to remove excess EdU and subsequently fixed in 4% paraformaldehyde (PFA) in ASW for 30 mins at r.t. Animals were then rinsed several times in phosphate buffer saline (PBS) and subsequently exposed to 1X PBT (1X PBS + 0.1% Triton X-100) before visualization of EdU. EdU incorporation was visualized using the Click-iT EdU Alexa Fluor 488 Imaging kit (Invitrogen) following the manufacturer’s instructions and a nuclear counterstain of 0.125 µg/ml Hoescht 33322 was used at 1:300 at r.t. for 10 mins. The number of EdU^+^ nuclei and the proportion of EdU^+^ nuclei across stages 3–6 were quantified and statistical significance tested.

In addition, 45-min EdU labeled animals were also used for immunostaining with phospho-histone (PH3) (1:1000) antibody to further confirm cell proliferation profiles. Immunostaining was performed following previously established protocols in *C. teleta* [1, 2]. Following immunostaining, the EdU incorporation was visualized using the Click-iT AlexaFluor 488 Imaging Kit (Invitrogen).

1. **EdU pulse-chase labeling**

In order to track fate and migration of dividing cells animals were incubated in 3 µM EdU in ASW for 30 min followed by chases in 30 µM thymidine for 2 h and subsequent incubation in sea-water until different lengths of time. EdU pulses were conducted at late stage 4 (both brain and VNC neurogenesis ongoing) in order to track cell movements in both brain and VNC [2]. As the duration of transition between each stage of larval development is 20-24 h, chases were conducted for 3 h, 6 h, 9 h, 12 h, 20 h, 36 h and 48 h time points following 30’ EdU incorporation in order to assay the cell division patterns of EdU^+^ cells within stages of larval development. After chases for respective time lengths in sea-water, the animals were fixed and the Click-iT EdU reaction (Invitrogen) was performed following the manufacturer’s instructions. Animals were cleared in 80% glycerol in PBS and imaged using a Zeiss IP-Apotome M2 with an AxioCam Mrm camera. EdU-pulse chase experiments and counting were conducted across three biological replicates i.e. using offspring from three different mothers. We sampled six offspring from each of the three mothers per time-point post-EdU pulse (n = 48 per mother in total). Altogether, 144 animals were sampled across all timepoints and all replicates.

1. **BrdU pulse-chase-wait-EdU sequential labeling**

Further sequential labelling using 5-Bromo-2′-deoxyuridine (BrdU) in combination with EdU was used to detect cells that are undergoing multiple rounds of divisions. For EdU/BrdU dual labeling, stages 4 and 5 animals were exposed to 0.1 mg/ml BrdU for 2 h and chased with 30 µM thymidine for another 3 h. Subsequently, animals were allowed to develop in sea-water until 24 h and 48 h before exposing them to 3 µM EdU for 2 h followed by pretreatment in 1:1 0.37 mol/L MgCl_2_:ASW for 5 mins and fixation in 4% PFA:ASW for 30 mins at r.t. EdU/BrdU dual pulse labelling was conducted by modifying existing protocols described in *C. teleta* [3] and elsewhere [4]. Following fixation, animals were washed in PBS and digested with 0.01 mg/mL Protienase K (Invitrogen #25530049) for 3 mins. Remaining Proteinase K was washed off using a series of PBS washes and animals were re-fixed in 4% PFA:PBS for 10 mins at r.t. Following washing of the fixative, DNA was denatured by incubating animals in pre-warmed 4 mol/L HCl for 15–30 mins at 37°C. The solution was then neutralized using 5–7 washes 0.1 mol/L sodium borate over 30 mins. Animals were then washed in PBS and subsequently exposed to PBS + 0.5% Triton X-100, followed by the Click-iT EdU reaction (Invitrogen) using the manufacturer’s instructions. After EdU detection, animals were blocked for immunostaining (PBT + 10% goat serum; Sigma G9023) for 1 h at r.t. Animals were then incubated overnight at 4°C in mouse anti-BrdU antibody (3D4) (1:100; BD Biosciences cat# 555627) diluted in block solution. Following 1° antibody incubation, animals were rinsed in PBT followed by 4–5 PBT washes of 20–30 min each and subsequently incubated in donkey anti-mouse-Alexa546 2° antibody (1:100; Invitrogen cat# A21245) as well as 0.125 µg/mL Hoescht 33322 (Invitrogen) overnight at 4°C. Following 2° antibody incubation, animals were rinsed twice in PBT and washed 4–5 times in PBT at 4°C. Animals were then equilibrated in 90% glycerol in PBS before imaging.

**Fluorescent In-Situ Hybridization (FISH) coupled to simple EdU incorporation**

Animals exposed to 3 µM EdU incorporation for 30 minutes were fixed for in situ hybridization [5, 6] and then dehydrated into 100 % methanol as described previously [5]. FISH was conducted against SoxB1 homologs (*Ct-soxB1*), proneural homologs (*Ct-ash1*, *Ct-ngn*), and neural differentiation markers (*Ct-neuroD*, *Ct-elav1*) [6] using protocols for non-fluorescent in situ hybridization but with minor modifications [5, 7]. Larvae were exposed to 0.01 mg/ml Proteinase-K for a shorter time, 2 mins, at room temperature. After hybridization, weaker riboprobes (*Ct-ash1*, *Ct-elav1*) were washed in less stringent washes, 0.2X SSC, instead of 0.05X SSC as for the other riboprobes (*Ct-soxB1*, *Ct-ngn* and *Ct-neuroD*). Digoxigenin-labeled riboprobes were generated using the MEGAscript kit (Ambion) and used at a working concentration of 1–2 ng/µl. SSC washes were followed by two 5 min washes with TNT buffer (0.1M Tris-HCl, pH 7.5, 0.15M NaCl, 0.1% Tween 20). After a series of washes in PBTx (1X PBS + Triton X-100 + 5% BSA), animals were blocked in freshly prepared Boehringer-Mannheim Blocking Reagent diluted in maleic acid and subsequently incubated in anti-digoxygenin-POD 1° antibody (1:500) overnight at 4°C. For fluorescent detection of probes, animals were first graduated into 1X amplification buffer (0.1M Boric acid, pH = 8.0 and 0.003% H_2_O_2_) and then exposed to the Cy3-NEN Tyramide Signal Amplification (TSA™) Plus reagent (Perkin Elmer) in amplification buffer (1:50). The fluorescent signal was allowed to develop for 3-4 h or longer at room temperature. The amplification reaction was stopped with successive 1X PTw (PBS + 0.1% Tween-20) washes after which the Click-iT EdU reaction was performed. Animals were incubated in 0.125 µg/mL Hoechst 33342 (Invitrogen) for at least 15–20 mins at room temperature and cleared in 80% glycerol in PBS or SlowFade Gold (Invitrogen) before analysis.

**Double fluorescent in-situ hybridization (dFISH)**

Animals were fixed for WMISH and hybridized with both Digoxigenin and Fluorescein labeled probes against the aforementioned genes in different combinations. Digoxigenin-labeled riboprobes were used at a final concentration of 1 ng/µl whereas Fluorescein-labeled riboprobes were used at a final concentration of 2 ng/µl. However weaker probes (e.g. *Ct-ash1*, *Ct-elav1*) were used at higher concentrations pertaining to the requirement of the experiment. Hybridization at 65°C were followed by stringent SSC washes using 2X and 0.2X SSC. For double-FISH, the animals were incubated in two different antibodies one at a time. Animals were incubated first in the anti-fluorescein-POD 1° antibody (1:250) in Boehringer-Mannheim Blocking Reagent overnight. Fluorescence from the fluorescein riboprobes were then detected using the Alexa-488-NEN Tyramide Signal Amplification (TSA™) Plus reagent (Perkin Elmer) in amplification buffer (1:25). The fluorescent signal was allowed to amplify for 5-6 h at room temperature until it reached desirable intensities. Following amplification of the fluorescein probe, the digoxygenin probe was developed using the Cy3-NEN Tyramide Signal Amplification (TSA™) Plus reagent (Perkin Elmer) in amplification buffer (1:50). Once desirable staining was obtained, animals were washed in 1X PTw at r.t. and incubated in 0.125 µg/mL Hoechst 33342 (Invitrogen) prior to equilibration in 80% glycerol in PBS or SlowFade Gold (Invitrogen).

**Supplementary Tables:**

**Table S1: Marginal and conditional R^2^ values for mixed effects models estimated for number of EdU and Hoescht labeled cells and proportion of cells labeled with EdU in the head and trunk segments for EdU-pulse chase experiments**

| Part | Model | R^2^m | | R^2^c |
| --- | --- | --- | --- | --- |
| Head | EdU counts | 0.808 | 0.950 | |
| Head | Hoescht counts | 0.735 | 0.962 | |
| Head | Proportion of EdU | 0.635 | 0.881 | |
| Trunk | EdU counts | 0.848 | 0.978 | |
| Trunk | Hoescht counts | 0.883 | 0.982 | |
| Trunk | Proportion of EdU | 0.455 | 0.856 | |

**Table S2: Percent variance explained by each random effect out of the total variance explained by all random effects for EdU pulse chase experiments**

| Part | Model | Mother | Individual | Layer/ segment | Side |
| --- | --- | --- | --- | --- | --- |
| Head | EdU counts | 14.79 | 1.30 | 57.89 | 26.02 |
| Head | Hoescht counts | 25.46 | 0.00 | 60.21 | 14.32 |
| Head | Proportion of EdU | 9.16 | 1.11 | 57.22 | 32.51 |
| Trunk | EdU counts | 28.81 | 43.15 | 15.75 | 12.29 |
| Trunk | Hoescht counts | 44.78 | 25.50 | 14.48 | 15.24 |
| Trunk | Proportion of EdU | 24.64 | 27.64 | 21.23 | 26.49 |

***Note:** Individual is nested within mother

Layer/segment applies to head/trunk, respectively, and is nested within individual within mother

**Table S3: Marginal and conditional R^2^ values for mixed effects models estimated for number of EdU and Hoescht labeled cells and proportion of cells labeled with EdU in the trunk segments for static 30-min EdU labeling and FISH+EdU experiments**

| Part | Model | R^2^m | | R^2^c |
| --- | --- | --- | --- | --- |
| Trunk | EdU counts | 0.625 | 0.938 | |
| Trunk | Hoescht counts | 0.847 | 0.923 | |
| Trunk | Proportion of EdU | 0.393 | 0.849 | |
| Trunk | *Ct-soxB1*^+^/EdU^+^ counts | 0.603 | 0.868 | |
| Trunk | *Ct-soxB1*^+^/EdU^+^ proportion | 0.387 | 0.567 | |
| Trunk | *Ct-ngn*^+^/EdU^+^ counts | 0.588 | 0.588 | |
| Trunk | *Ct-ngn*^+^/EdU^+^ proportion | 0.238 | 0.417 | |
| Trunk | *Ct-ash1^+^*/EdU^+^ counts | 0.672 | 0.766 | |
| Trunk | *Ct-ash1^+^*/EdU^+^ proportion | 0.662 | 0.750 | |

**Table S4: Percent variance explained by each random effect out of the total variance explained by all random effects for static 30-min EdU labeling and FISH+EdU experiments**

| Part | Model | Individual | Segment | Side |
| --- | --- | --- | --- | --- |
| Trunk | EdU counts | 69.87 | 13.62 | 16.51 |
| Trunk | Hoescht counts | 35.21 | 14.81 | 49.98 |
| Trunk | Proportion of EdU | 61.87 | 13.28 | 24.86 |
| Trunk | *Ct-soxB1*^+^/EdU^+^ counts | 66.99 | 0.00 | 33.01 |
| Trunk | *Ct-soxB1*^+^/EdU^+^ proportion | 29.41 | 0.00 | 70.59 |
| Trunk | *Ct-ngn*^+^/EdU^+^ counts | 0.00 | 0.00 | 100.00 |
| Trunk | *Ct-ngn*^+^/EdU^+^ proportion | 23.53 | 0.00 | 76.47 |
| Trunk | *Ct-ash1^+^*/EdU^+^ counts | 28.71 | 0.00 | 71.29 |
| Trunk | *Ct-ash1^+^*/EdU^+^ proportion | 26.09 | 0.00 | 73.91 |

***Note:** Segment is nested within individual

**Table S5: Kinetics of EdU^+^/gene^+^ cells in the anterior neuroectoderm**

|  | EdU^+^/Ct-soxB1^+^ | | EdU^+^/Ct-ngn^+^ | | EdU^+^/Ct-ash1^+^ | |
| --- | --- | --- | --- | --- | --- | --- |
| Stage | Counts ± S.E.M. | Proportion ± S.E.M. | Counts ± S.E.M. | Proportion ± S.E.M. | Counts ± S.E.M. | Proportion ± S.E.M. |
| **ST3** | 25.5 ± 2.848 | 0.397 ± 0.037 | 17.3 ± 1.520 | 0.229 ± 0.022 | 6.1 ± 1.222 | 0.077 ± 0.013 |
| **ST4** | 49.3 ± 2.403 | 0.524 ± 0.013 | 31.5 ± 2.093 | 0.280 ± 0.019 | 12.6 ± 1.050 | 0.143 ± 0.0145 |
| **ST5** | 60.3 ± 1.943 | 0.418 ± 0.025 | 44.5 ± 2.473 | 0.281 ± 0.013 | 17.3 ± 2.360 | 0.138 ± 0.017 |
| **ST6** | 41.4 ± 2.541 | 0.365 ± 0.026 | 29.2 ± 2.136 | 0.265 ± 0.016 | 20.6 ± 2.731 | 0.179 ± 0.018 |

**Table S6: Kinetics of EdU^+^/gene^+^ cells in the trunk neuroectoderm**

|  |  | EdU*^+^*/*Ct-soxB1^+^* | | EdU*^+^*/*Ct-ngn^+^* | | EdU*^+^*/*Ct-ash1^+^* | |
| --- | --- | --- | --- | --- | --- | --- | --- |
|  | Stage | Counts ± S.E.M. | Proportion ± S.E.M. | Counts ± S.E.M. | Proportion ± S.E.M. | Counts ± S.E.M. | Proportion ± S.E.M. |
| ROI 1 | **ST4** | 11.4 ± 0.858 | 0.752 ± 0.020 | 5.4 ± 0.476 | 0.245 ± 0.021 | 0.4 ± 0.163 | 0.0338 ± 0.014 |
|  | **ST5** | 20.3 ± 1.584 | 0.655 ± 0.034 | 12.5 ± 1.910 | 0.339 ± 0.047 | 7.4 ± 0.791 | 0.163 ± 0.015 |
|  | **ST6** | 26.7 ± 3.791 | 0.914 ± 0.032 | 16.3 ± 1.429 | 0.438 ± 0.072 | 4.5 ± 1.017 | 0.095 ± 0.023 |
| ROI 2 | **ST4** | 10.7 ± 0.955 | 0.764 ± 0.029 | 10.0 ± 0.557 | 0.445 ± 0.027 | 0.0 ± 0.000 | 0.000 ± 0.000 |
|  | **ST5** | 21.1 ± 2.126 | 0.730 ± 0.053 | 11.2 ± 1.451 | 0.350 ± 0.041 | 6.9 ± 0.604 | 0.199 ± 0.02 |
|  | **ST6** | 40.0 ± 3.675 | 0.887 ± 0.032 | 23.0 ± 2.633 | 0.399 ± 0.057 | 7.1 ± 1.287 | 0.106 ± 0.018 |
| ROI 3 | **ST6** | 52.6 ± 2.339 | 0.825 ± 0.026 | 25.0 ± 1.949 | 0.415 ± 0.06 | 8.7 ± 0.977 | 0.110 ± 0.012 |

**References**:

1. Meyer NP, Carrillo-Baltodano A, Moore RE, Seaver EC: **Nervous system development in lecithotrophic larval and juvenile stages of the annelid Capitella teleta**. *Front Zool* 2015, **12**:15.

2. Meyer NP, Seaver EC: **Neurogenesis in an annelid: characterization of brain neural precursors in the polychaete Capitella sp. I**. *Dev Biol* 2009, **335**(1):237-252.

3. de Jong DM, Seaver EC: **Investigation into the cellular origins of posterior regeneration in the annelid Capitella teleta**. *Regeneration (Oxf)* 2018, **5**(1):61-77.

4. Bradford JA, Clarke ST: **Dual-pulse labeling using 5-ethynyl-2'-deoxyuridine (EdU) and 5-bromo-2'-deoxyuridine (BrdU) in flow cytometry**. *Curr Protoc Cytom* 2011, **Chapter 7**:Unit 7 38.

5. Seaver EC, Paulson DA, Irvine SQ, Martindale MQ: **The spatial and temporal expression of Ch-en, the engrailed gene in the polychaete Chaetopterus, does not support a role in body axis segmentation**. *Dev Biol* 2001, **236**(1):195-209.

6. Sur A, Magie CR, Seaver EC, Meyer NP: **Spatiotemporal regulation of nervous system development in the annelid Capitella teleta**. *Evodevo* 2017, **8**:13.

7. Seaver EC, Kaneshige LM: **Expression of 'segmentation' genes during larval and juvenile development in the polychaetes Capitella sp. I and H. elegans**. *Dev Biol* 2006, **289**(1):179-194.
